# Bioinspired Cellulose‐Based Ultra‐Slippery Film with Superior Transmittance, Anti‐Fouling and De‐Icing Properties for the Durable and Efficient Output of Solar Panels

**DOI:** 10.1002/advs.202514626

**Published:** 2025-10-03

**Authors:** Hujun Wang, Chuangqi Mo, Xueping Zhang, Jing Zheng, Gaohui Han, Haonan Qiu, Bo Li, Kai Yin, Zhongrong Zhou

**Affiliations:** ^1^ Tribology Research Institute State Key Laboratory of Rail Transit Vehicle System School of Mechanical Engineering Southwest Jiaotong University Chengdu 610031 P. R. China; ^2^ Key Laboratory of Bionic Engineering (Ministry of Education) Jilin University Changchun 130022 P. R. China; ^3^ Hunan Key Laboratory of Nanophotonics and Devices School of Physics Central South University Changsha 410083 P. R. China

**Keywords:** bioinspired design, de‐icing, optical transparency, slippery surface, solar panel

## Abstract

High optical transmittance can endow solar panels with sufficient light energy intake, while anti‐fouling and anti‐icing properties ensure stable power generation in environments where dust, bird droppings, algae, and ice are prone to accumulate. A highly transparent and ultra‐slippery surface is promising for meeting these requirements. However, it remains a huge challenge to achieve superior transmittance, anti‐fouling, anti‐icing, and durability on the same surface to ensure high energy conversion efficiency for solar panels. Herein, a bioinspired cellulose‐based ultra‐slippery film (BCUSF) with an extremely low water sliding angle (SA = 0.4°) and high transmittance (≈95% of bake glass) is reported. Benefiting from the impressive slippery property, remarkably low ice adhesion strength (0.38 kPa), and superior self‐cleaning and anti‐fouling performances are also demonstrated. Moreover, the BCUSF exhibits excellent durability and robustness, maintaining a SA of 0.8° after suffering high shear at 9000 r min^−1^. Accordingly, the BCUSF with highly comprehensive performance enables solar panels to maintain high energy‐conversion efficiency after repeated accumulation/cleaning of ice (ice adhesion strength = 0.91 kPa after 25 tests) and dust, or sand impact. It is envisioned that the BCUSF can boost the practical applications of slippery films on solar panels.

## Graphical abstract

Accumulations of ice, dust, bird droppings, and algae pose a significant risk of reducing the energy‐conversion efficiency of solar panels. Here, an articular cartilage‐inspired cellulose‐based ultra‐slippery film with highly comprehensive performances is reported. Specifically, the bioinspired film integrates superior optical transparency, anti‐fouling and de‐icing properties, which can ensure the durable and efficient power generation of solar panels.

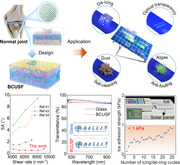

## 1 Introduction

Clean energy holds utmost significance in our pursuit of a sustainable future, as it plays a vital role in combating climate change and reducing environmental contamination. Solar panels are key devices that convert clean, renewable solar energy into usable electricity for daily life and industrial production.^[^ ^1^ ^]^ However, icing seriously affects the performance and safety of solar panels.^[^ ^2^, ^3^ ^]^ Daily accumulations of dust and bird droppings, and algae propagation in warm and humid regions, lead to a decrease in energy‐conversion efficiency.^[^ ^4^ ^]^ To address these issues, an attractive approach is the development of multifunctional high‐transparency surfaces with excellent anti‐icing, self‐cleaning, and anti‐fouling properties.

Superhydrophobic phenomenon is ubiquitous in nature, with lotus leaves being a typical example.^[^ ^5^, ^6^ ^]^ Over the past two decades, researchers have developed various superhydrophobic surfaces to meet the aforementioned requirements because of their super‐low adhesion to water and ice.^[^ ^7^, ^8^, ^9^ ^]^ However, the micro/nanostructures determining superhydrophobic performance tend to interlock with ice under low‐temperature conditions, which increases ice adhesion strength and thereby limits practical applications.^[^ ^10^, ^11^, ^12^ ^]^ Fortunately, nature offers abundant inspiration for the development of advanced artificial systems. Inspired by the *Nepenthes* pitcher plant, slippery liquid‐infused porous surfaces (SLIPSs) have been proposed,^[^ ^13^ ^]^ which can separate ice from substrates through a layer of lubricant, overcoming the anti‐icing shortcomings of superhydrophobic surfaces.^[^ ^14^, ^15^, ^16^ ^]^ However, the lubricant on SLIPSs is easily lost after a few icing/de‐icing cycles.^[^ ^17^, ^18^ ^]^ Although lubricants can be re‐infused, it is highly desirable to enhance the stability of the lubrication layer for minimizing lubricant consumption and associated costs.

In the early stages, a SLIPS substrate should have micro/nanotextures to result in an infused lubrication layer stabilized by capillary forces. However, the resulting lubrication layer shows moderate stability. Many strategies have emerged to delay the failure of the lubrication layer. Among them, liquid‐ and solid‐like slippery surfaces without micro/nanotextures can enhance the stability of lubrication layers by replacing liquid lubricants, but generally with an increase in ice adhesion strength.^[^ ^19^, ^20^, ^21^ ^]^ For self‐replenishing slippery surfaces without micro/nanotextures, the lost lubricant can be replenished by gel matrices, but insufficient mechanical durability and adhesive strength of the elastic gels limit their applications.^[^ ^22^, ^23^ ^]^ It is evident that developing a slippery surface with integrated excellent slippery and anti‐icing performances, superior durability and robustness, and strong adhesion strength to substrates remains challenging. Moreover, the effect on energy‐conversion efficiency must be considered when creating a slippery surface on solar panels. Therefore, a slippery surface with highly comprehensive performance should be developed to assist solar panels in achieving high operating efficiency under various harsh environments.

Here, we considered the lubricating property of articular cartilage, which involves the fixation of water lubrication layers by the hydrophilic lubrication complexes anchored on the surface of the cartilage,^[^ ^24^, ^25^ ^]^ to prepare a bioinspired cellulose‐based ultra‐slippery film (BCUSF). Unlike most previous reports that used epoxy resin or polydimethylsiloxane (PDMS) as the matrix of slippery films, our BCUSF was composed of a biodegradable ethyl cellulose (EC) matrix, PDMS, and silicone oil (SO). The EC matrix is not only environmentally friendly, but also facilitates the grafting of reactive PDMS due to its abundant hydroxyl groups, thereby enhancing the adsorption of lubricant SO. The BCUSF exhibited an extremely low sliding angle (SA, SA = 0.4°) and a remarkably low ice adhesion strength (0.38 kPa). Importantly, the film showed good optical transparency (transmittance >87%) in the wavelength range between 450 and 950 nm, and thereby had a minimal effect on the energy‐conversion efficiency of solar panels. We also demonstrated the durability, robustness, and strong resistance to the accumulation of dust, bird droppings, and algae. Finally, we showed that the BCUSF allowed solar panels to maintain high conversion efficiency after repeated accumulation/cleaning of ice and dust, or sand impact. In particular, the ice adhesion strength only increased to 0.91 kPa after 25 tests on the BCUSF‐coated solar panels. This study confirmed the significant potential of slippery surfaces for ensuring the normal operation of solar panels in harsh environments.

## 2 Results and Discussion

### 2.1 Design and Fabrication

**Figure 1:**
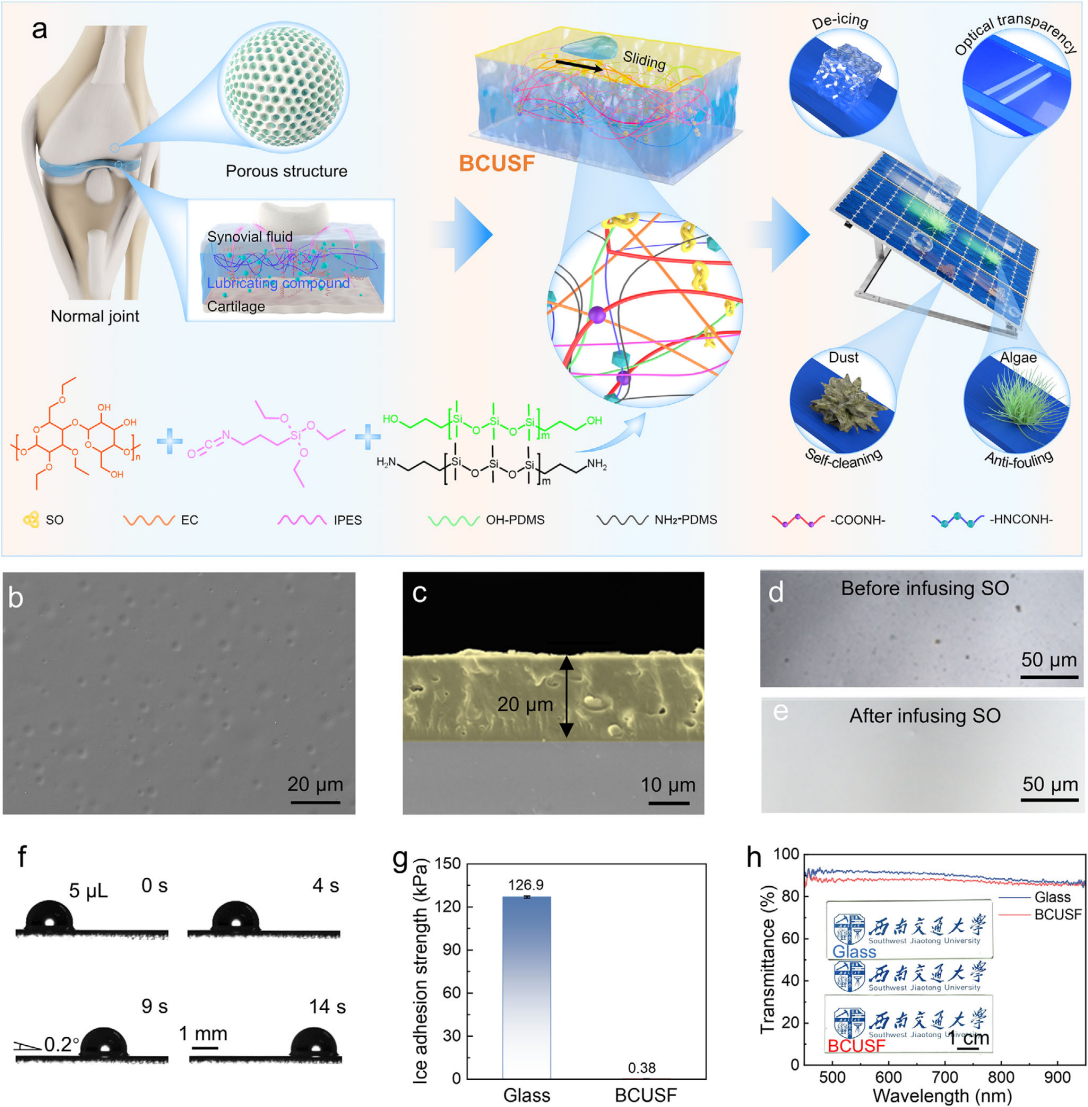
Design and characterizations. a) Schematic diagram for the design of the BCUSF. SEM images of the b) surface and c) longitudinal section of the EINO film. LSCM images of the BCUSF d) before and e) after infusing SO. f) Images showing the sliding process of a 5 µL water droplet on the BCUSF. g) Ice adhesion strength of bare glass sheets and BCUSFs. h) Transmittance spectra of bare and BCUSF‐coated glass sheets. The inset shows a bare and BCUSF‐coated glass sheet.

Healthy natural cartilage exhibits a very low friction coefficient of ≈0.001 to ≈0.03, which is closely related to the surface structures and chemical compositions.^[^ ^26^ ^]^ The micropores on the cartilage surface can store synovial fluid, which contributes to the cartilage lubrication.^[^ ^27^ ^]^ The hydrophilic lubrication complexes anchored on the cartilage surface adsorb water, forming a stable hydrated layer to reduce friction.^[^ ^24^, ^28^ ^]^ To enhance the stability of the lubrication layer of slippery liquid‐infused surfaces, we designed a BCUSF with surface microstructures to store lubricants and anchoring molecules capable of bridging the lubrication layer and film matrix (**Figure** **1**a). Unlike most previous reports that used epoxy resin or polydimethylsiloxane (PDMS) as the matrix of slippery films, biodegradable ethyl cellulose (EC) was chosen as the matrix of our BCUSF due to its environmental friendliness, multiple chemical binding sites, superior film‐forming ability, high optical transparency, and high adhesive strength to substrates. 3‐isocyanatopropyltrimethoxysilane (IPES) was grafted onto EC through the reaction of the N═C═O of IPES with the ─OH of EC to form EI (Figure S1, Supporting Information).^[^ ^29^ ^]^ The N═C═O of excess IPES reacted with the ‐NH_2_ of amino‐terminated PDMS (NH_2_‐PDMS) to form NI.^[^ ^29^ ^]^ Under the catalysis of dibutyltin diacetate (DBTDA), hydroxyl‐terminated PDMS (OH‐PDMS) and NI were grafted onto EC through the reaction of the siloxane group with the ─OH.^[^ ^30^, ^31^ ^]^ Finally, silicone oil (SO) can be adsorbed onto the EC matrix through the van der Waals force between the PDMS and SO, thus forming a stable lubrication layer.^[^ ^32^, ^33^ ^]^ The IPES, NH_2_‐PDMS, and OH‐PDMS acted as anchoring molecules.

According to the above design concept, the BCUSF was prepared (Figure S2, Supporting Information). First, EC, IPES, NH_2_‐PDMS, DBTDA, and OH‐PDMS were sequentially dissolved into ethyl acetate (EA) with an interval between each addition of the reagents, which enabled the occurrence of specific chemical reactions. Then, the solution was spin‐coated onto glass substrates and solidified to form a solid film (EINO). Finally, SO was infused into the film to obtain a BCUSF. The influence of the chemical components on the slippery property was investigated. It was found that the films were unable to achieve superior slippery performance without the use of IPES for the chemical grafting of PDMS onto EC (Figure S3a, Supporting Information). The EINO film showed high adhesion to water droplets (SA = 90°), demonstrating the importance of SO infusion. Excessive PDMS resulted in increased SA due to the high roughness of the film surface (Figure S3b,c, Supporting Information). The optimal slippery performance was obtained when the ratio of NH_2_‐PDMS to OH‐PDMS was 0:1 (Figure S3d,e, Supporting Information). To obtain better wear resistance, a modest amount of NH_2_‐PDMS (NH_2_‐PDMS:OH‐PDMS = 1:3) was added, which was discussed later.

Figure S4 (Supporting Information) shows the changes in chemical functional groups during the film formation process. For pure EC film, the peak at 3476 cm^−1^ corresponded to ‐OH,^[^ ^34^ ^]^ serving as the reaction site for IPES grafting. For IPES, the characteristic peak at 2270 cm^−1^ was attributed to N═C═O.^[^ ^35^ ^]^ Upon mixing EC with IPES, the peaks of N═C═O and ─OH in the resultant EI film disappeared, accompanied by the emergence of new peaks at 3335 cm^−1^ (─NH─), indicating the reaction between ─OH in EC and N═C ═O in IPES to form NH─COO.^[^ ^36^ ^]^ In comparison, a stronger peak appeared at 1633 cm^−1^ (C═O) in the EIN film formed by mixing EC, IPES, and NH_2_‐PDMS, suggesting the formation of NH─CO─NH.^[^ ^35^ ^]^ Compared to EIN, the addition of OH─PDMS resulted in the enhancement of Si─O─Si stretching (1015 cm^−1^) in the formed EINO film.^[^ ^37^ ^]^

Figure 1b,c shows the SEM images of the as‐prepared EINO film, which possessed randomly distributed micropits and a thickness of ≈20 µm. The formation of the micropits may be related to the local aggregation of the NH_2_‐PDMS and OH‐PDMS, because the positions of micropits corresponded to Si‐rich regions (Figure S5, Supporting Information). As shown in Figure 1d,e, a continuous and smooth lubrication layer was formed on the film surface after infusing SO into the EINO film. To confirm the infusion of SO in the film, we added Nile Red to SO, followed by examining the cross‐section of the BCUSF. To avoid the transfer of oil from the top to the cross‐section, the film should not be cut from the top to the bottom. Fluorescence image demonstrated the successful infusion of SO (Figure S6, Supporting Information). The thickness (≈55 nm) of the SO overlayer was estimated as described in the experimental section because it is difficult to distinguish the thin layer using the inverted microscope. Figure 1f shows an extremely low average SA (0.4°) of the BCUSF, enabling a 5 µL water droplet to slide on the surface with an extremely low tilt angle (Movie S1, Supporting Information). To demonstrate the de‐icing capability of the BCUSF, the ice adhesion strength of the bare and BCUSF‐coated glass sheets was measured. It can be seen from Figure 1g that the BCUSF endowed the glass sheet with an extremely low ice adhesion strength of 0.38 kPa, whereas a high ice adhesion strength of 126.9 kPa was observed on the bare glass sheet. Figure 1h shows a high optical transparency of the BCUSF. The blue logo, Chinese characters and letters underneath the BCUSF‐coated glass sheet were very clear. In the wavelength from 450 to 950 nm, the transmittance of the BCUSF (>87%) slightly decreased by ≈5% when compared with that of the bare glass slide. The results suggested that in the event of ice formation on the surface of solar panels, the ice on the BCUSF‐coated surface can be easily removed, thereby maintaining a high conversion efficiency of the solar panels. By contrast, the efficiency of original solar panels would sharply decrease due to the difficulty in removing accumulated ice.

### 2.2 Slippery Property, Durability, and Robustness

**Figure 2:**
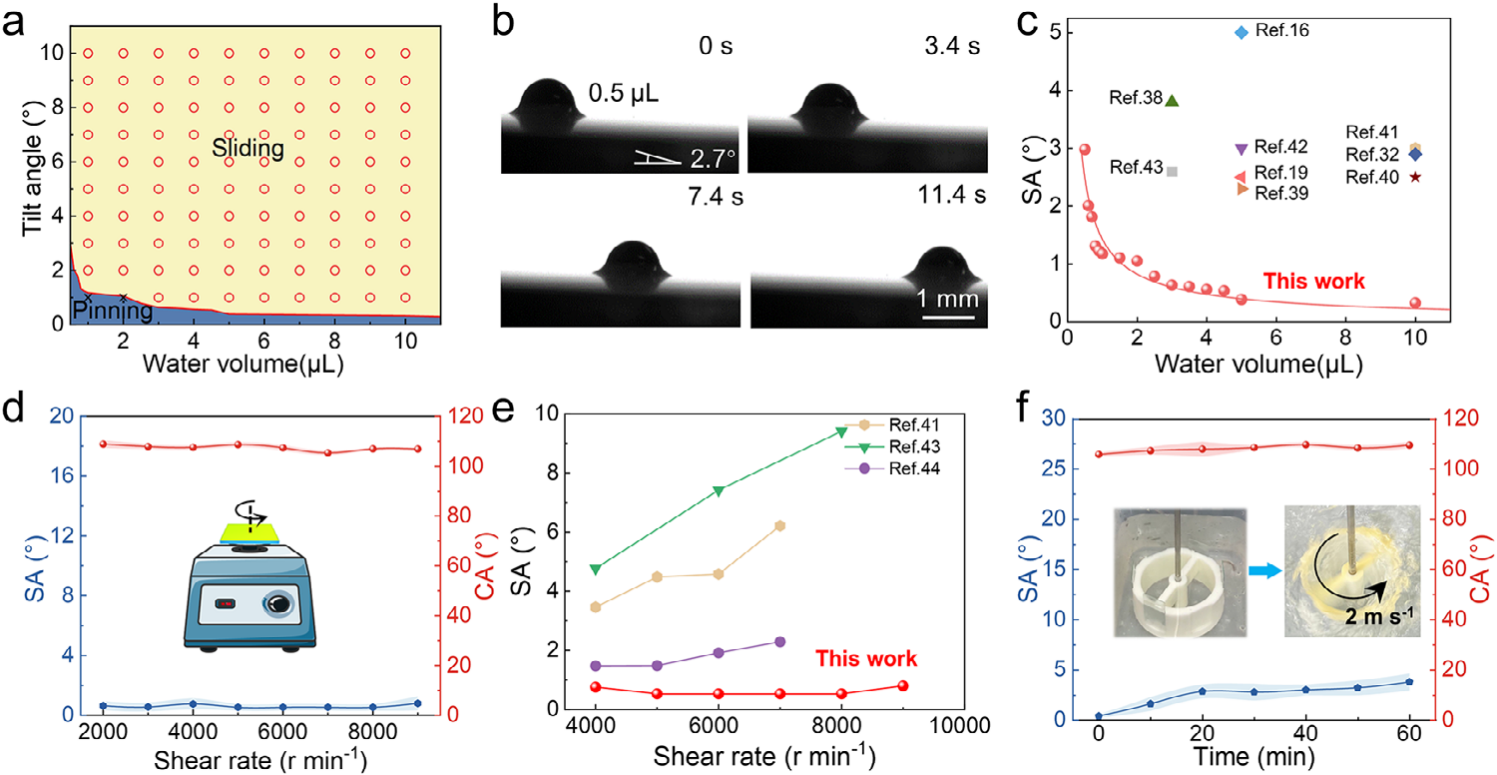
Slippery property, durability, and robustness. a) Effect of droplet volume and the tilt angle of BCUSF‐coated surfaces on droplet sliding. b) Selected images of the sliding process of a 0.5 µL water droplet on a BCUSF. c) Comparison of the slippery property of BCUSF with that of previously reported materials. d) Effect of shear rate used to control centrifugal force on the SA of the BCUSF. e) Comparison of the resistance of the BCUSF to high‐speed shear with that of the existing reports. f) Effect of rotation time in water on the SA of the BCUSF.

We then performed systematic tests to demonstrate the slippery property of the BCUSF (**Figure** **2**a). It was found that when the volume of water droplets equaled or exceeded 2.5 µL, the SA was less than 1°. Even for a tiny droplet of 0.5 µL, easy sliding was also observed on the BCUSF (Figure 2b, Movie S2, Supporting Information). The superiority of the BCUSF over previous state‐of‐art slippery surfaces was shown in Figure 2c, which confirmed the excellent slippery property of our BCUSF.^[^ ^16^, ^19^, ^32^, ^38^, ^39^, ^40^, ^41^, ^42^, ^43^ ^]^ To achieve such slippery property, PDMS needed to be grafted onto EC via IPES acting as a bridge, followed by the strong adsorption of SO onto the film surface through PDMS. As shown in Figure S3a (Supporting Information), films lacking IPES or PDMS exhibited poor slippery property.

The strong adsorption of the lubrication layer to the film surface was crucial for maintaining the slippery property under harsh conditions. Figure 2d shows the resistance of the BCUSF to strong shear (Movie S3, Supporting Information). Even after high‐speed shear at 9000 r min^−1^ for 3 min, the BCUSF still exhibited a low SA of 0.8°. The shear resistance of the film was superior to reported slippery liquid‐infused surfaces (Figure 2e).^[^ ^41^, ^43^, ^44^ ^]^ Figure 2f shows the variation in SA of the BCUSF after rotation in water. The rotational speed and linear velocity were 350 r min^−1^ and 2 m s^−1^, respectively. The BCUSF retained a good slippery property with a small SA of 3.8° after undergoing 60 min of water shearing.

We then tested the durability of the BCUSF when encountering water‐jet impact, long‐term storage, high temperature, UV irradiation, soaking in acid and alkali. Figure S7a (Supporting Information) shows the resistance of the BCUSF to water‐jet impact at a speed of 3 m s^−1^. After being impacted for 120 s, the BCUSF showed a low SA of 3.8°, and the dyed water droplets could easily slide off without leaving any residue (Movie S4, Supporting Information). The SA of the BCUSF showed a slight change, increasing to 2.2° after being stored in air for 6 months (Figure S7b, Supporting Information). When the heating temperature was increased to 120 °C, the BCUSF showed a slight change in SA (SA = 1.2°, Figure S7c, Supporting Information), which was sufficient for its application on solar panels. As shown in Figure S7d (Supporting Information), UV irradiation (250 W, 365 nm) had no obvious effect on the slippery property of the BCUSF. After immersing the BCUSF in aqueous solutions with pH = 0 (SA = 1.0°) or pH = 14 (SA = 1.7°) for 12 h, very low SA was observed (Figure S7e,f, Supporting Information). As shown in Figure S8 (Supporting Information), the loss rate of the SO was provided after the tests including high‐speed shear (9000 r min^−^^1^, 3 min), rotation in water (350 r min^−1^, 60 min), high temperature (120 °C, 10 min), and water‐jet impact (3 m s^−^^1^, 120 s). Due to the harsh test conditions, a high loss rate of the SO was observed. Nonetheless, the retained SO still ensured a good slippery property of the BCUSF.

In addition to the stable adsorption of the lubrication layer, we further analyzed other mechanisms underlying the exceptional slippery performance of the BCUSF. When a water droplet was deposited on the oil‐infused surface, the oil might spread over and cloak the droplet, leading to oil losses as the droplet slid off the surface (Figure S9, Supporting Information). The criterion for cloaking behaviors was given by the spreading coefficient in the air environment, *S*_ow(a)_ ≡ *γ*_wa_ – *γ*_ow_ – *γ*_oa_, where *γ* represented the interfacial tension between the two phases designated by subscripts w (water), o (oil), and a (air).^[^ ^45^ ^]^ *S*_ow(a)_ > 0 implied the occurrence of cloaking, whereas *S*_ow(a)_ < 0 implied otherwise. According to Girifalco‐Good theory, *γ*_ow_ = *γ*_wa_ + *γ*_oa_ – 2*φ*(*γ*_wa_ *γ*_oa_)^1/2^. For the liquids of water and oil, γ_wa_ = 72.8 mN m^−1^, γ_oa_ = 22.4 mN m^−1^, *φ* = 0.5, the calculated *S*_ow(a)_ = −4.4 mN m^−1^. Consequently, the water droplet on the BCUSF was not cloaked by oil. When the water droplet contacted the surface, it was also important whether the lubrication layer was replaced by water, which was evaluated by the spreading coefficient in the water environment, *S*_os(w)_ ≡ *γ*_oa_cos *θ*_os(a)_ – *γ*_wa_cos *θ*_ws(a)_ – *γ*_ow_, where subscript s represented solid.^[^ ^45^ ^]^ As shown in Figure S10 (Supporting Information), *θ*_os(a)_ = 0°, *θ*_ws(a)_ = 118°, the calculated *S*_os(w)_ = 1.76 > 0. Thus, the lubrication layer on BCUSF was stable and would not be replaced by water.

**Figure 3:**
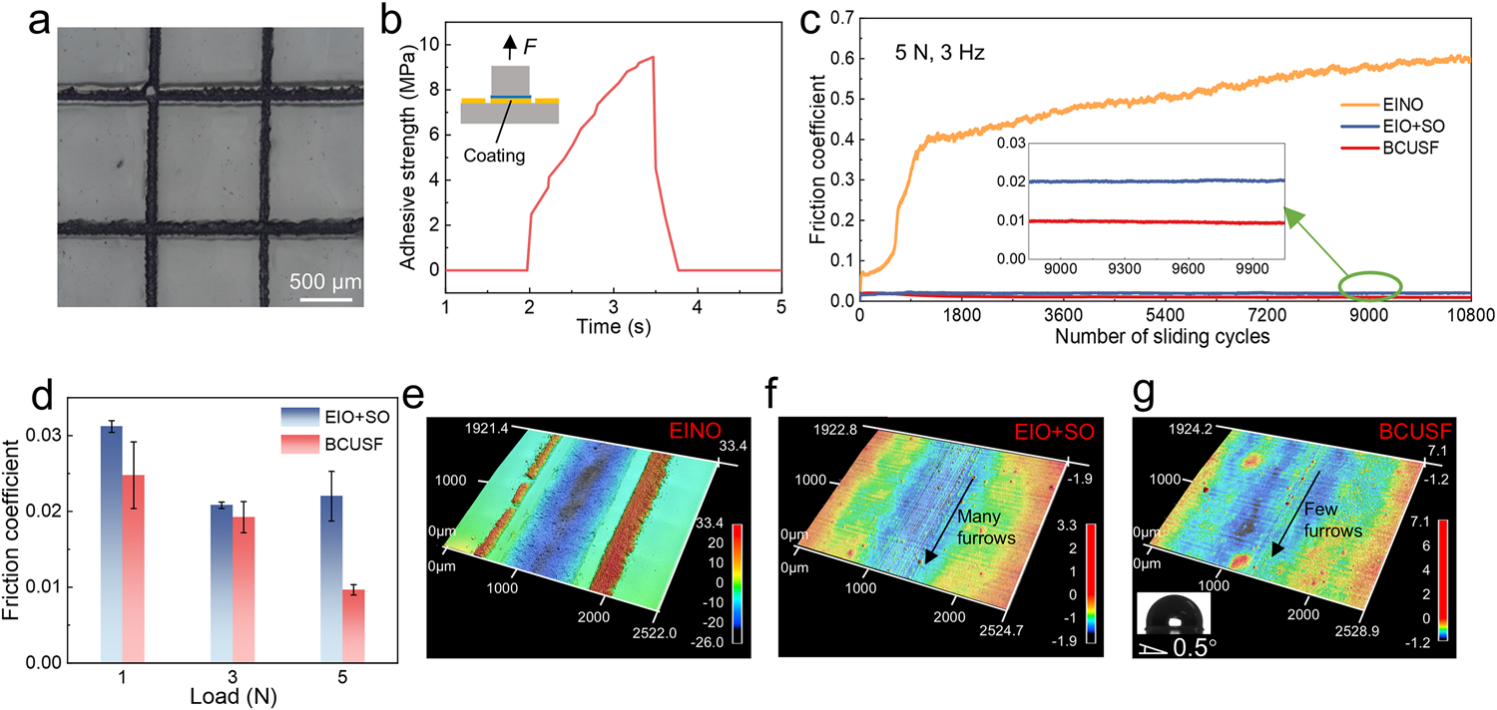
Mechanical robustness. a) Optical photograph of the EINO film after the cross‐cut test. b) Variation in the adhesive strength between the EINO film and glass substrate with time. c) Change in the friction coefficient of EINO, EIO+SO, and BCUSF with friction cycles at 5 N and 3 Hz. d) Effect of load on the average friction coefficient of EIO+SO and BCUSF. e–g) LSCM images after the friction tests corresponding to (c). The inset shows the SA of the BCUSF after wear.

The adhesive capability of films to substrates is a critical indicator of mechanical robustness. The adhesive performance of the EINO film was evaluated by the cross‐cut test according to the ISO 2409 standard at first. Regular grids with an interval of 1 mm were created on the coated glass sheet. 3 M tape was applied onto the damaged film surface and then peeled off. We observed that no apparent fragments peeled off and the grids maintained a complete square, indicating that the adhesion met the 0 standard (**Figure** **3**a). The value of adhesive strength was also measured according to the ISO 4624 standard. Figure 3b shows a high adhesive strength (9.5 MPa) of the film. The adhesion stability of films after exposure to alternating high and low temperatures was a critical parameter for evaluating their long‐term reliability in practical environments. To evaluate this property, the film was heated in an oven at 60 °C for 2 h, followed by cooling in a freezer at −20 °C for 2 h. After 30 such cycles, the adhesive strength only decreased to 9.3 MPa (Figure S11, Supporting Information).

The mechanical robustness was characterized by friction and wear tests. As shown in Figure 3c, because of the infusion of SO, the BCUSF exhibited a low and stable friction coefficient of 0.01 after 10 000 cycles. The NH_2_‐PDMS also contributed to the low friction coefficient because it provided greater elasticity than OH‐PDMS, as evidenced by the lower elastic modulus of EINO (2.6 ± 0.2 GPa) relative to EIO (EINO without NH_2_‐PDMS, 3.07 ± 0.17 GPa). This was confirmed by the lower friction coefficient of the BCUSF under a high load (Figure 3d). Generally, materials with higher elasticity have greater deformation under high loads, which leads to the extrusion of more lubricants. Therefore, the friction coefficient of the BCUSF continuously decreased with increasing load, and the friction coefficient of the SO‐infused EIO film (EIO+SO) did not decrease as the load increased from 3 to 5 N. From Figure 3e–g, a slight wear scar was observed on the worn BCUSF, illustrating the superior wear resistance. Moreover, the worn BCUSF showed a slight change in SA because of its negligible wear and the strong self‐repairing capability of slippery liquid‐infused surfaces.

### 2.3 De‐Icing, Self‐Cleaning, and Anti‐Fouling Properties

This paper focused on the de‐icing performance of our films, as ice accumulation markedly reduced the efficiency of solar panels, hindering their safety operation. Although traditional mechanical, chemical, and thermal de‐icing technologies are effective, they suffer from drawbacks such as low efficiency, environmental pollution, and high energy consumption.^[^ ^46^, ^47^, ^48^ ^]^ Especially for mechanical de‐icing, the high adhesion strength of ice makes it challenging to achieve complete removal without damaging the surface of solar panels. If the ice adhesion strength was significantly reduced, mechanical de‐icing of solar panels would become highly feasible. The extremely low SA of the BCUSF indicated its excellent water‐repellent property, which resulted from the ability of the lubrication layer to separate water from the substrate. As a result, after the freezing of water, the non‐frozen lubrication layer reduced the adhesion strength of the ice to the substrate, making it easier to be mechanically removed (Figure S12a, Supporting Information).

**Figure 4:**
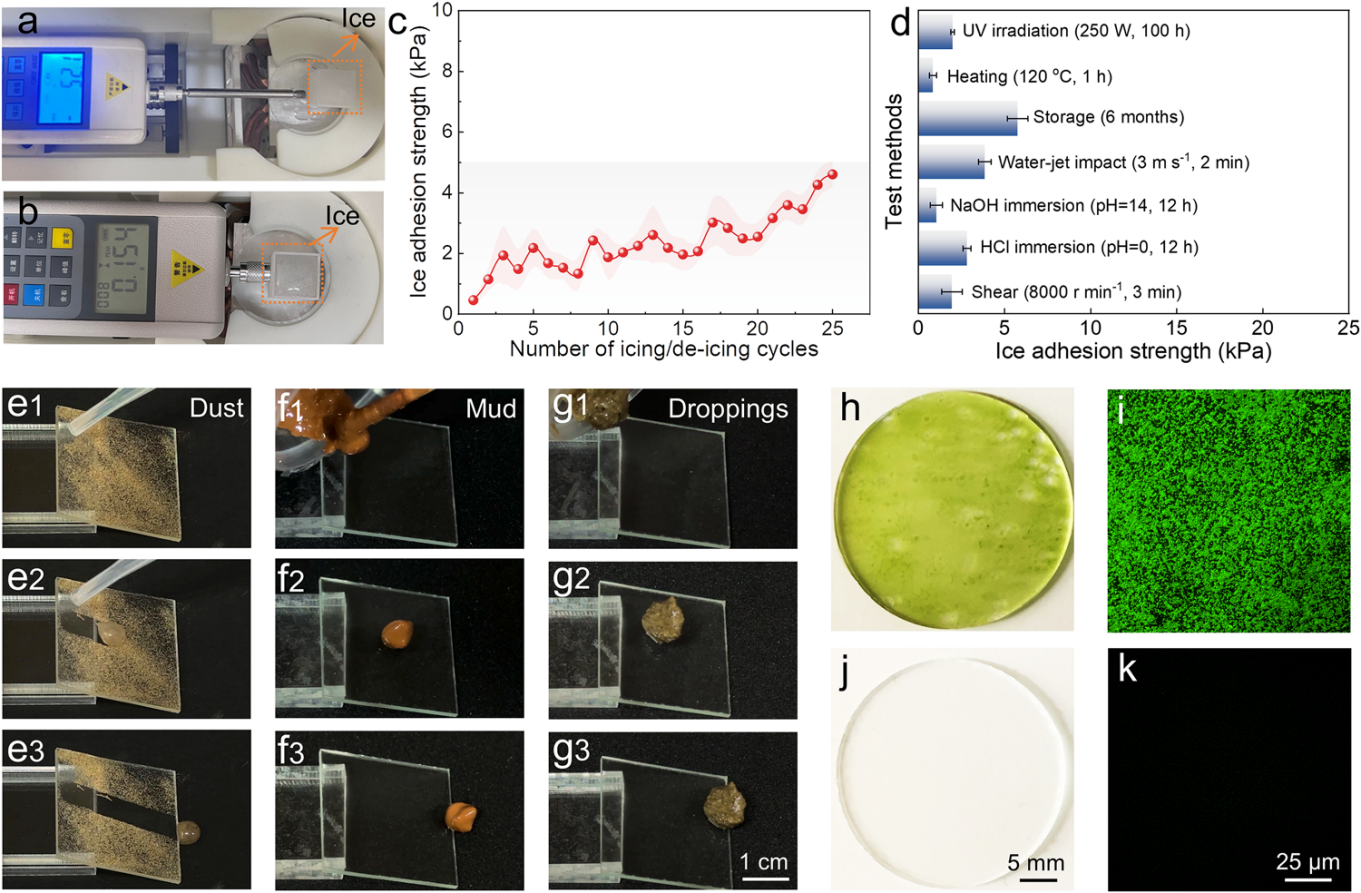
De‐icing, self‐cleaning, and anti‐fouling properties. Optical photographs showing the push force required for the ice column to detach from a) bare and b) BCUSF‐coated glass sheets. c) Variation in adhesion strength of ice on bare and BCUSF‐coated glass sheets with icing/de‐icing cycles. d) Summary of the ice adhesion strength on the BCUSF after different tests. e–g) Sequential images of the self‐cleaning processes to mimic dust, mud, and bird droppings. h) Optical photograph and i) corresponding fluorescence image of the bare glass sheet after being placed in chlorella solution for 7 days. j) Optical photograph and k) corresponding fluorescence image of the BCUSF‐coated glass sheet after being placed in chlorella solution for 7 days.

For the bare glass sheet, the average push force required for the ice column to detach from the surface was 50.8 N, and the average ice adhesion strength was 126.9 kPa (**Figure** **4**a, Movie S5, Supporting Information). On our film, an average push force of only 0.15 N was needed to detach the ice column, demonstrating an impressively low ice adhesion strength (average value = 0.38 kPa, Figure 4b, Movie S6, Supporting Information). This implied that mechanical de‐icing was efficient and feasible for the BCUSF‐coated solar panels. The low ice adhesion strength was primarily attributed to the lubrication layer separating the ice from the substrate, as confirmed by the observation that the EINO film exhibited a high ice adhesion strength (66 kPa, Figure S12b, Supporting Information). Slippery liquid‐infused surfaces have the advantage of recovering the slippery performance by re‐infusing lubrication oil after its loss. Nevertheless, to minimize maintenance frequency of solar panels and reduce lubricant consumption, it is imperative for such surfaces to maintain a low ice adhesion strength even after multiple icing/de‐icing cycles. As shown in Figure 4c, the stable lubrication layer allowed the film to possess a low ice adhesion strength of <5 kPa after enduring 25 icing/de‐icing cycles. The increase in ice adhesion strength was likely attributed to the unavoidable leakage of water from the cube and the condensation of atmospheric moisture, leading to the formation of a thin ice layer outside the test region. The ice layer might accelerate the loss of SO. As the test region expanded, the service life of the lubrication layer was prolonged, which was subsequently confirmed by the durable de‐icing performance of BCUSF‐coated solar panels. Moreover, even after undergoing shear at 8000 r min^−1^ for 3 min, soaking in strong acid or alkali for 12 h, water‐jet impact at 3 m s^−1^ for 120 s, storage for 6 months, heating at 120 °C for 60 min, or UV irradiation for 100 h, the BCUSFs showed a low ice adhesion strength of < 6 kPa (Figure 4d). The results demonstrated an excellent de‐icing performance of our BCUSFs.

To investigate the resistance of the film to other pollutants, we conducted self‐cleaning and anti‐fouling tests. Figure S13 (Supporting Information) shows the resistance of the BCUSF to contamination of various liquids. As shown in Figure 4e, because of the slippery property, dust was enwrapped and removed by water droplets sliding off from the BCUSF surface (Movie S7, Supporting Information). We prepared mud using 50 wt.% red clay and 50 wt.% water, and observed their sliding behaviors on the film surface under gravity. The BCUSF exhibited good resistance to the contamination by mud, and it also demonstrated a good repellency to bird droppings (60 wt.% of water and 40 wt.% pigeon droppings) (Figure 4f,g, Movies S8 and S9, Supporting Information). Notably, the bare glass sheet and superhydrophobic surface were contaminated by the mud with high viscosity (Figure S14, Supporting Information). Furthermore, the investigation of the propagation of chlorella on the bare and BCUSF‐coated glass sheets was performed. For the uncoated glass sheet, 22.1% area was covered by chlorella after 1 day of culture, and the coverage area reached 47.7% after a culture time of 7 days (Figure 4h,i and Figure S15, Supporting Information). The accumulation of algae on solar panels not only decreased the energy‐conversion efficiency but also caused a potential risk of damaging the solar panels. Additionally, the algae adhering to the rough structures of solar panel surfaces were difficult to be completely removed. In sharp contrast, almost no chlorella (day 7, coverage area = 0.87%) was detected on the BCUSF‐coated glass sheet due to the ultra‐low adhesion of the BCUSF to chlorella and the physical separation by the lubrication layer, as shown in Figure 4j,k and Figure S15 (Supporting Information). These results clarified the enormous potential of the BCUSF for de‐icing, self‐cleaning, and anti‐fouling applications.

### 2.4 Application on Solar Panels

**Figure 5:**
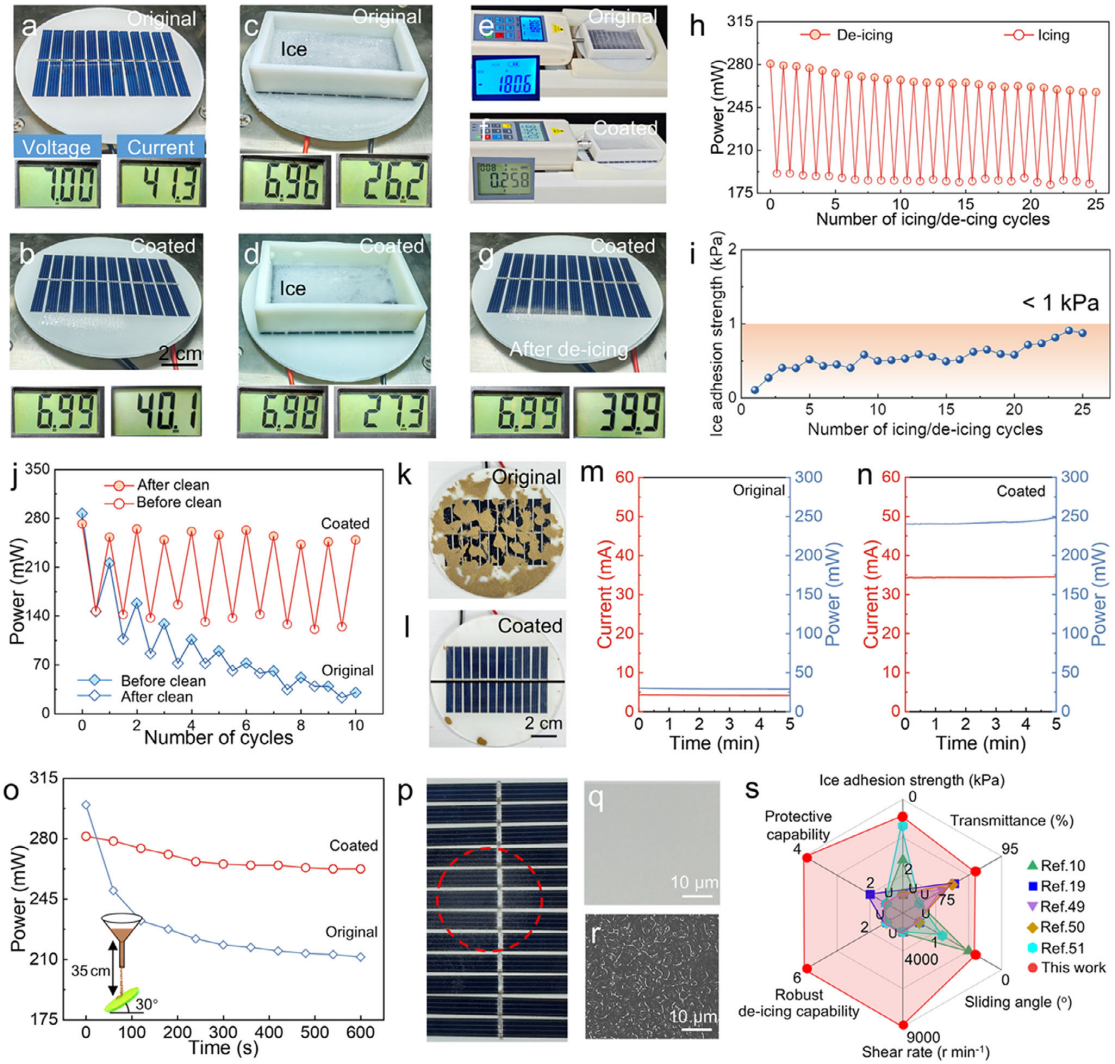
Protective capability for solar panels. Optical photographs showing the voltage and current of a) original and b) BCUSF‐coated solar panels. Images of the voltage and current of the c) original and d) BCUSF‐coated solar panels after icing. e) Image showing the difficulty of ice detachment from the original solar panel. f) Image showing the easy ice detachment from the BCUSF‐coated solar panel. g) Image showing the voltage and current of the BCUSF‐coated solar panel after de‐icing. Variations in h) output power and i) critical push force for ice detachment on BCUSF‐coated solar panels with icing/de‐icing cycles. j) Variation in output power of the original and BCUSF‐coated solar panels with dust contamination/cleaning cycles. Images of the k) original and l) BCUSF‐coated solar panels after 10 cycles of repeated contamination and cleaning. Changes in output power and current of the m) BCUSF‐coated and n) original solar panels with time for cycle 10. o) Variation in output power of the original and BCUSF‐coated solar panels with sand impact time. p) Optical photographs of a BCUSF‐coated solar panel after sand impact for 600 s. LSCM images of the BCUSF‐coated solar panel q) before and r) after sand impact for 600 s. s) Radar map of the comprehensive performance of the BCUSF compared with that of previously reported materials. “U” represents that the studies did not provide the corresponding data or that the provided values are outside the specified range. The numbers corresponding to protective capability and robust de‐icing capability represent the number of tolerable destructive events.

Given the de‐icing, self‐cleaning, and anti‐fouling performance of the transparent BCUSF, we demonstrated its application in the protection of solar panels. As shown in **Figure** **5**a,b, the output power of the original solar panel was 289.1 mW, which slightly decreased to 280.3 mW after applying the BCUSF. When the original and BCUSF‐coated solar panels were covered by ice, the output power sharply decreased to 182.4 mW and 190.6 mW, respectively (Figure 5c,d). Because of the strong adhesion between the ice and the original solar panel, the ice could not be removed even if the push force was increased to 180.6 N (Figure 5e, Movie S10, Supporting Information). Although increasing the force was beneficial for ice removal, it raised the risk of damaging the overall structure of solar panels in practical applications. Because the ice was not removed, the solar panel had to operate with a low output power. In sharp contrast, the ice on the BCUSF‐coated solar panel was easily removed under a low push force of 0.26 N (Figure 5f, Movie S11, Supporting Information). Accordingly, the output power was restored to 278.9 mW with the removal of the ice (Figure 5g). More importantly, the BCUSF endowed the solar panel with a stable output power of 257.6 mW after 25 icing/de‐icing cycles (Figure 5h). Notably, although the critical push force slightly increased with increasing the number of icing/de‐icing cycles, it was far less than that needed for the removal of the ice from the original solar panel (Figure 5i). The ice adhesion strength was measured to be as low as 0.91 kPa during the 25th de‐icing test. This was due to that 22% of the SO was still held by BCUSF (Figure S16, Supporting Information).

Accumulation of dust has an obvious effect on the energy‐conversion efficiency of solar panels. We compared the resistance to dust accumulation of two solar panels: one was coated with the BCUSF, and the other was original. Dust (1 g) was scattered onto the solar panels to simulate dust accumulation, and the panels were then cleaned by mimicking rainfall. As shown in Figure S17 (Supporting Information), when the two samples were placed at a tilt angle of 30° for 1 h, 1 g of dust still adhered to the surfaces without visible detachment. We used a small volume of water (5 mL) to clean the surfaces, reflecting the potential water scarcity in arid regions. After 1 cycle of contamination and cleaning, the BCUSF‐coated solar panel retained a high output power (253.0 mW) because of the self‐cleaning effect of the BCUSF, whereas the output power of the original solar panel sharply decreased to 215.8 mW (Figure 5j). After 10 cycles, a large amount of dust accumulated on the original solar panel, whereas the BCUSF‐coated solar panel remained clean (Figure 5k,l). Therefore, the BCUSF‐coated solar panel maintained an output power of 249.2 mW, which was eight times higher than that of the original solar panel with dust accumulation (29.3 mW, Figure 5m,n).

Accumulation of pollutants such as ice and dust generally does not cause the permanent damage of solar panels. However, sandstorms can easily damage solar panels, making them rough and leading to a permanent decrease in energy‐conversion efficiency. Consequently, we investigated the protective capability of the BCUSF against sandstorms by allowing falling sands to impact the original and BCUSF‐coated solar panels. As shown in Figure 5o, the output power of the original solar panel decreased to 249.9 mW after being subjected to falling sand for 60 s. By increasing the impact time of falling sands to 600 s, the output power dramatically decreased to 211.4 mW, which was 70.6% of the original solar panel's output power. For the BCUSF‐coated solar panel, after exposure to falling sand for 60 s, the output power decreased to 278.6 mW. As the sand impacting time prolonged, the output power gradually decreased at first and then tended to stabilize. The output power of the BCUSF‐coated solar panel after impact for 600 s was 262.5 mW, which was 87.6% of that of the original solar panel before sand impact. Figure 5p shows slight impact marks on the surface of the BCUSF‐coated solar panel. From the LSCM images, a certain degree of wear was observed on the BCUSF, and the continuous oil layer was damaged (Figure 5q,r). However, the resulting rough surface still retained a small amount of lubrication oil, which aided in light transmission. As shown in Figure S18 (Supporting Information), it was found that the original solar panel suffered from serious wear. Obviously, surface wear markedly reduced light transmittance, thereby decreasing the energy conversion efficiency. As shown in Figure S19 (Supporting Information), the tilt angle of the solar panels and the height of the funnel from the solar panels also affected the change in energy‐conversion efficiency. We emphasized that the BCUSF always provided strong protection for the solar panels against sand impact.

To evaluate BCUSF performance, we selected the following indicators for comparison (Figure 5s). The SA, allowable maximum shear rate before SA > 1°, ice adhesion strength, and transmittance were considered in the comparison. Robust de‐icing capability under harsh conditions was also demonstrated, which was indicated by the low ice adhesion strength (< 6 kPa) after undergoing shear at 8000 r min^−1^, soaking in strong acid or alkali, water‐jet impact at 3 m s^−1^, storage for 6 months, heating at 120 °C, or UV irradiation (power of UV lamp = 250 W). Moreover, we showed the protective capability of the BCUSF for solar panels to resist the ice, dust, and algae, as well as sand impact. For the aforementioned metrics, the BCUSF outperformed previously reported materials,^[^ ^10^, ^19^, ^49^, ^50^, ^51^ ^]^ allowing it to be applied effectively on solar panels.

Finally, we discussed the anti‐aging property, economic feasibility, and scalability of the BCUSF. According to the international standard of ISO 4892, the aging test was performed by using xenon arc lamps to simulate sunlight (1200 W m^−2^, 168 h) at 40 °C, equivalent to 3 months of Sichuan natural sunshine. The SA of the BCUSF‐coated solar panel only increased to 1.3° (Figure S20a, Supporting Information), which was consistent with the increase in SA under heating conditions (Figure S7c, Supporting Information). The output power slightly decreased by 2.3% compared to the original BCUSF‐coated solar panel (Figure S20b, Supporting Information). The ice adhesion strength increased from 0.1 to 0.14 kPa (Figure S20c, Supporting Information). As listed in Table S1 (Supporting Information), the cost ($1.69 m^−2^) of the film raw materials was provided. Moreover, we fabricated BCUSF‐coated solar panels by the roller coating method to demonstrate the scalability for large‐area coatings. Compared to the BCUSF prepared by the spin coating method on solar panels, the BCUSF fabricated via the roller coating method achieves a nearly unchanged SA and output power, and a slightly increased ice adhesion strength to 0.12 kPa (Figure S21, Supporting Information). The above characteristics of the BCUSF confirm its potential for application on solar panels.

## 3 Conclusions

In summary, we developed an ultra‐slippery film that was inspired by the phenomenon of articular cartilage fixing lubricating water layers through the hydrophilic lubrication complexes anchored on the cartilage surface. The film exhibited an extremely low SA of 0.4° and a good optical transparency. Because of the grafting of PDMS to EC, SO was firmly adsorbed on the film surface, endowing the film with a stable lubrication layer. Therefore, the obtained BCUSF exhibited excellent durability and robustness. Notably, the film remained a very low SA of 0.8° even after being subjected to high shear at 9000 r min^−1^. The film showed a remarkably low ice adhesion strength of 0.38 kPa, which remained < 6 kPa even after high‐speed shear, soaking in strong acid or alkali, strong water‐jet impact, long‐term storage, high‐temperature heating, or high‐intensity UV irradiation. Moreover, the resistance of the film to the accumulations of dust, mud, bird droppings, and algae was demonstrated. Owing to its excellent performance across several key metrics, the film allowed solar panels to maintain a high energy‐conversion efficiency after repeated accumulation/cleaning cycles of ice and dust, or sand impact. Notably, the ice adhesion strength on the BCUSF‐coated solar panels was as low as 0.91 kPa after 25 cycles of icing/de‐icing tests. These properties will promote the application of slippery films on solar panels and other optical surfaces.

## 4 Experimental Section

### Materials and Sample Preparation‐Materials

EC (9.6 mPa·s in viscosity) was purchased from Shanghai Aladdin Biochemical Technology Co., Ltd. IPES was purchased from Nanjing Capatue Chemical Co., Ltd. DBTDA was purchased from Shanghai Yien Chemical Technology Co., Ltd. OH‐PDMS, NH_2_‐PDMS, and SO (10 cSt in viscosity) were purchased from Shenzhen Jipeng Silicon Fluoride Materials Co., Ltd. EA was purchased from Chengdu Kelong Chemical Co., Ltd. Nile red was purchased from Dalian Meilun Biotech Co., Ltd. Chlorella suspension was purchased from Nanjing Haiersi Biotechnology Co., Ltd. Solar panels were obtained from Shenzhen Langhang Electronics Co., Ltd.

### Materials and Sample Preparation‐Fabrication of BCUSF

0.5 g of EC was dissolved in 3 g of EA, followed by magnetically stirring for 30 min. Then, IPES (0.6 g) was added to the solution and stirred for 30 min. After adding NH_2_‐PDMS (0.025 g) and stirring for 30 min, DBTDA (0.02 g) and OH‐PDMS (0.075 g) were dissolved and blended for 30 min. The as‐prepared paint was deposited on substrates by spin coating at 2000 r min^−1^ for 25 s, and then was cured at 60 °C for 12 h. Finally, the coated surfaces were immersed into SO for 12 h, followed by spin coating at 1500 r min^−1^ for 25 s to remove excess SO and form the BCUSF.

### Characterizations‐Characterization of Surface Morphology

Surface morphologies were characterized by scanning electron microscope (SEM, Apreo 2C, Thermo Scientific) and laser scanning confocal microscope (LSCM, VK‐X1000, Keyence).

### Characterizations‐Analysis of Chemical Composition

A Fourier transform infrared spectrophotometer (FTIR, Nicolet iS50, Thermo Fisher Scientific) was used to analyze the chemical compositions of films.

### Characterizations‐Analysis of SO Infusion

Nile red was dissolved in SO, and then the dyed SO was infused into the as‐prepared films. The infusion of SO was analyzed by observing the longitudinal section of the films using an inverted microscope (IX73, Olympus).

### Characterizations‐Estimation for the Thickness of the SO Overlayer

To estimate the thickness of the SO overlayer on the as‐prepared BCUSF, the weights (*W*_u_) of the BCUSF‐coated glass sheets were measured using a precision balance. Afterward, the SO overlayer was removed using filter paper, and the weights (*W*_t_) of the treated samples were recorded. The thickness of the SO overlayer can be estimated as *T* = (*W*_u_‐*W*_t_)/(*ρ×S*), where *ρ* was the density of the SO, and *S* represented the area of the BCUSF.

### Characterizations‐Assessment of Slippery Performance

CA and SA were measured using a contact angle meter (SDC200, Sindin). Generally, 5 µL of liquid droplets were used to test CA and SA, and the average values were obtained from 3–5 individual measurements at different positions for each sample. Because of the presence of wetting ridges, the CA of the slippery liquid‐infused surfaces was estimated as an approximate value, which was used to determine the trend of CA variations. SA measurements were more critical for characterizing slippery performance. For SA measurements, a droplet was placed on a horizontally positioned sample. Then, the sample was slowly titled to allow the droplet to slide off. The tilt angle at which the droplet started sliding was denoted as the SA.

### Characterizations‐Assessment of Durability and Robustness

High‐speed shear tests lasting 3 min were performed using a spin coater. For water‐jet impacting test, high‐speed water jet impacted the BCUSF‐coated samples at a speed of ≈3 m s^−1^. For rotation tests in water, BCUSF‐coated samples were fixed at the modified paddle of mechanical stirrer and rotated at a speed of 350 r min^−1^. In storage tests, BCUSF‐coated samples were stored in a chamber under constant temperature and humidity (25 °C, 54% RH). Thermal stability was tested by placing the BCUSF‐coated samples in a heating oven for 10 min. UV resistance was tested by exposing BCUSF‐coated samples to UV light source (365 nm, 250 W) 15 cm away from the samples. Film samples were immersed in HCl solution (pH = 0) and NaOH solution (pH = 14) to test chemical stability. The adhesive capability was evaluated according to international standards, namely ISO 2409 (cross‐cut test) and ISO 4624 (pull‐off test). Reciprocating ball‐on‐plate tribological tests were conducted using UMT tribometer (UMT‐TriboLab, Bruker). In the tests, 440C steel balls with a diameter of 10 mm were used to rub the BCUSF‐coated samples at a frequency of 3 Hz.

### Characterizations‐De‐icing Test

To create an ice column, pure water in a cube (20 mm × 20 mm × 20 mm) was applied in contact with a BCUSF‐coated sample at ‐20 °C. After freezing for 12 h, the sample was taken out and immediately placed on a ‐15 °C cooling stage. Subsequently, the ice was detached by a motion stage at a controlled speed of 5.5 mm s^−1^, with a g auge recording the force. The ice adhesion strength was calculated by *τ*_ice_ = *F*/*A*, where *F* was the maximum force required for the complete removal of the ice column with a base area of *A*.

### Characterizations‐Algae Adhesion Test

A chlorella suspension with a concentration of 6.0‐7.0×10^6^ cells mL^−1^ was added into cell culture plates. Then, the samples with the film facing upward were placed in the plates and cultured for 1 day or 7 days (25 °C, 2000–3000 lx).

### Characterizations‐Output Power Test

The voltage and current were tested by exposing the solar panels to a light source (20 000 lx), which was 7.5 cm from the panels. Then, the corresponding output power was calculated.

### Characterizations‐Falling Sand Impacting Test

To investigate the capability of the BCUSF to withstand sandstorms, samples were exposed to falling sand. During the test, silica sands were dropped to continuously impact the BCUSF‐coated solar panels. The output power of the solar panels was measured after every 60 s of sand impact.

### Characterizations‐Aging Test

A xenon arc lamp (1000 W) was used to simulate sunlight according to an international standard (ISO 4892). The distance between the BCUSF and the light source was adjusted until the irradiance on the BCUSF surface reached ≈1200 W m^−2^, at which point the surface temperature was ≈40 °C. The aging test lasted for 168 h.

### Characterizations‐Nano‐Indentation Measurement

With the help of a nano‐indenter (G200, KLA‐Tencor, USA), the elastic modulus was obtained by nano‐indentation tests in XP mode. During the tests, samples were evaluated using a Berkovich diamond tip with a radius of 20 nm and applying a maximum load of 10 mN, a maximum indentation depth of 1.4 µm, load duration of 30 s, peak load delay of 2 s. To estimate the elastic modulus, the Poisson's ratio was set at 0.2.^[^ ^52^ ^]^

## Conflict of Interest

The authors declare no conflict of interest.

## Supporting information



Supporting Information

Supplemental Movie 1

Supplemental Movie 2

Supplemental Movie 3

Supplemental Movie 4

Supplemental Movie 5

Supplemental Movie 6

Supplemental Movie 7

Supplemental Movie 8

Supplemental Movie 9

Supplemental Movie 10

Supplemental Movie 11

## Data Availability

The data that support the findings of this study are available from the corresponding author upon reasonable request.
