# Supplementary material for: Bioinspired Cellulose‐Based Ultra‐Slippery Film with Superior Transmittance, Anti‐Fouling and De‐Icing Properties for the Durable and Efficient Output of Solar Panels
Source: Adv Sci (Weinh). 2025 Oct 3;12(48):e14626. doi: 10.1002/advs.202514626 (PMC12752601; doi:10.1002/advs.202514626)
Supplement: Supplementary file 1 — Supporting Information [file ADVS-12-e14626-s007.docx]

Supporting Information

**Bioinspired Cellulose-Based Ultra-Slippery Film with Superior Transmittance, Anti-Fouling and De-Icing Properties for the Durable and Efficient Output of Solar Panels**

*Hujun Wang^a^, Chuangqi Mo^a^, Xueping Zhang^b^, Jing Zheng^a,*^, Gaohui Han^a^, Haonan Qiu^a^, Bo Li^b,*^, Kai Yin^c,*^, Zhongrong Zhou^a^*

^a^Tribology Research Institute, State Key Laboratory of Rail Transit Vehicle System, School of Mechanical Engineering, Southwest Jiaotong University, Chengdu 610031, People’s Republic of China

^b^Key Laboratory of Bionic Engineering (Ministry of Education), Jilin University, Changchun 130022, People’s Republic of China

^c^Hunan Key Laboratory of Nanophotonics and Devices, School of Physics, Central South University, Changsha 410083, People’s Republic of China

^*^Corresponding authors: Jing Zheng, jzheng168@home.swjtu.edu.cn; Bo Li, boli@jlu.edu.cn; Kai Yin, kaiyin@csu.edu.cn

Keywords: Bioinspired design, Slippery surface, De-icing, Optical transparency, Solar panel

**Supplementary Figures**

**
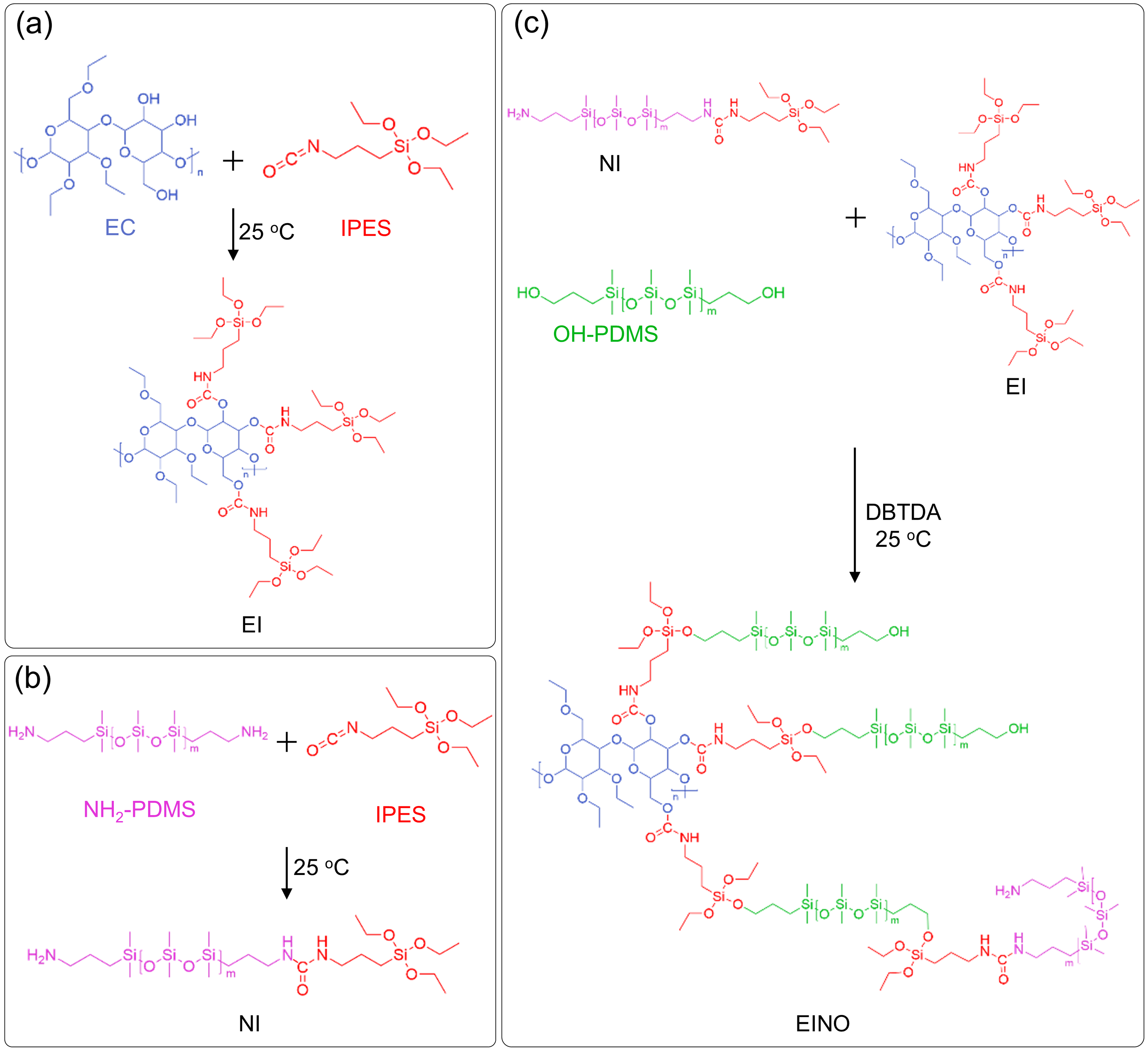
**

Figure S1. Schematic description of chemical grafting. Formation of (a) EI, (b) NI, and (c) EINO.


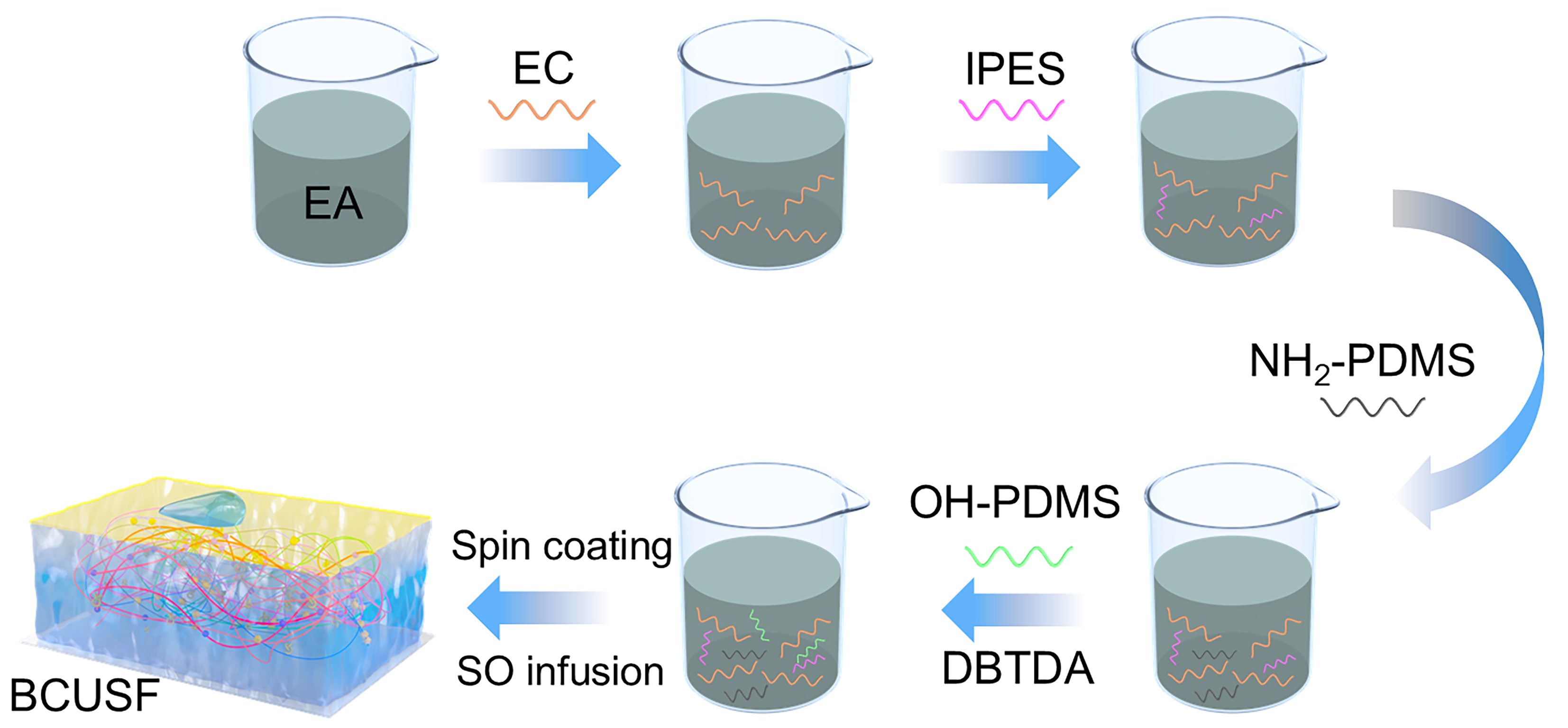


Figure S2. Schematic illustration of the fabrication of BCUSF.


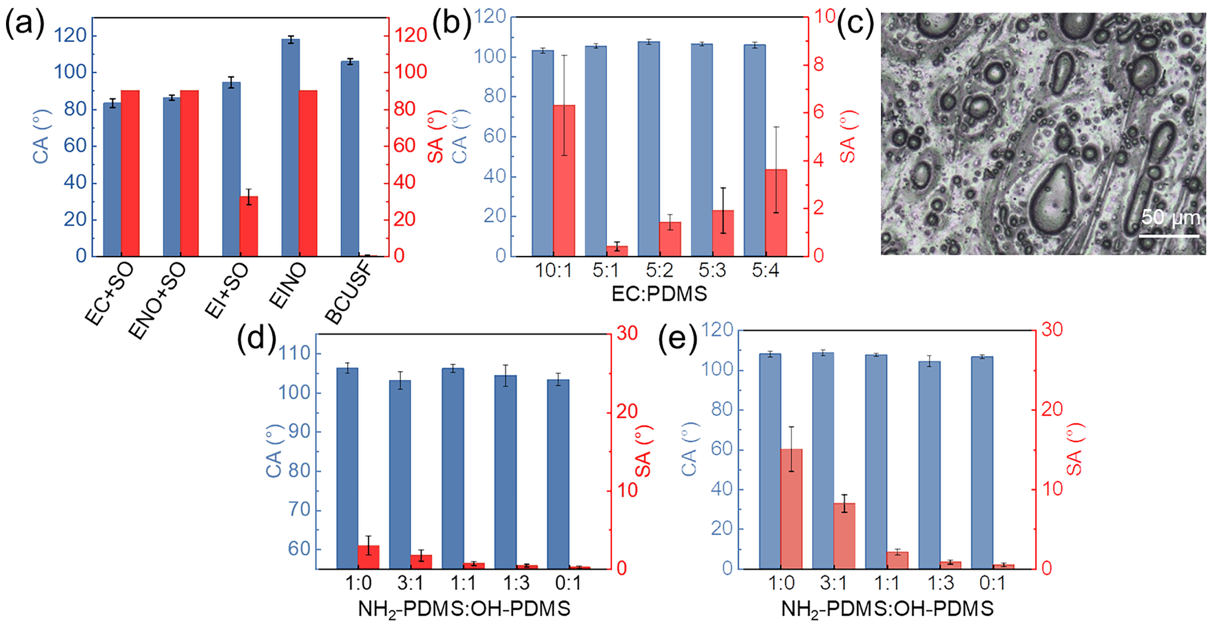


Figure S3. Effect of chemical components on wetting and slippery properties. (a) Comparison of various films. ENO represents the EINO without IPES (b) Effect of the content of PDMS (NH_2_-PDMS:OH-PDMS = 1:3). (c) LSCM image of BCUSF consisting of EC and PDMS in a 5:4 ratio. Effect of the ratio of NH_2_-PDMS and OH-PDMS on the performance of the BCUSF (d) before and (e) after undergoing shear at 8000 r/min for 3 min.


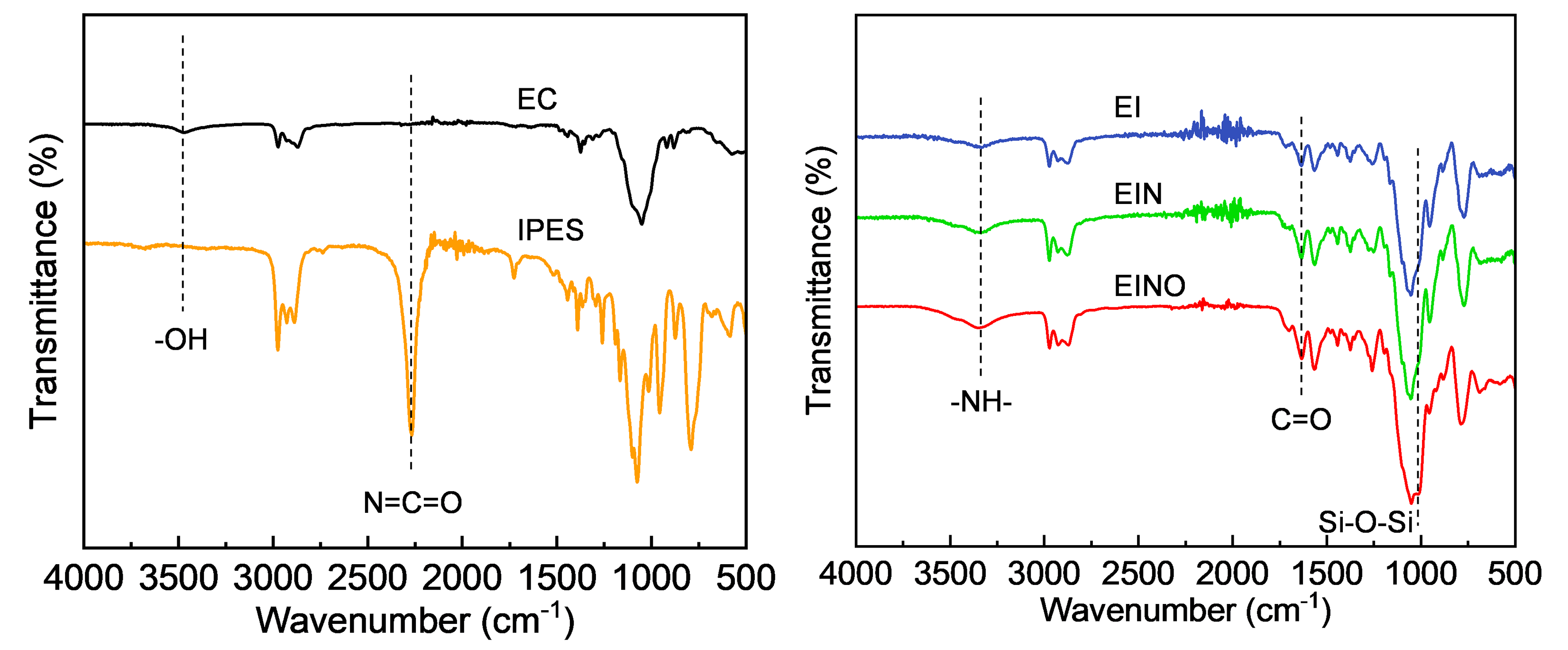


Figure S4. FTIR spectra of (a) EC, IPES, and (b) EI, EIN, and EINO.


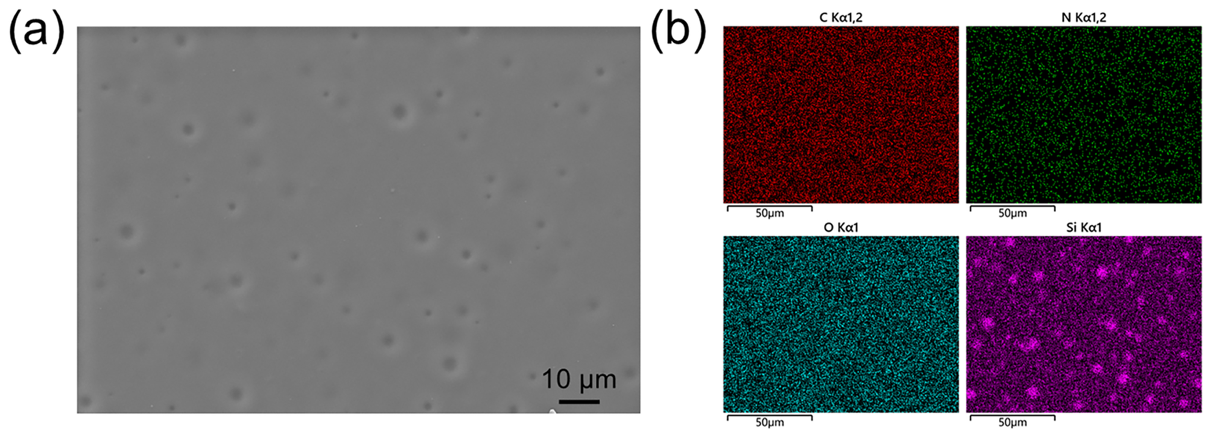


Figure S5. (a) SEM image and (b) the corresponding EDS mapping of the EINO film.


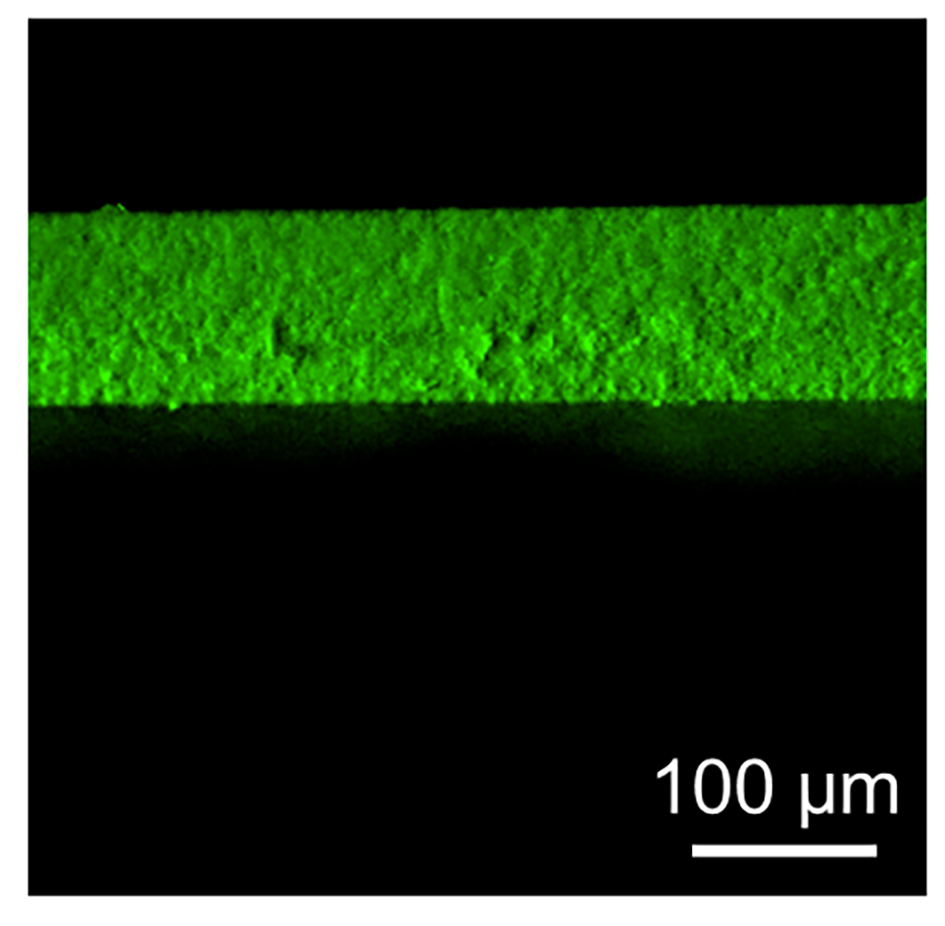


Figure S6. Fluorescence image of the longitudinal section of BCUSF.


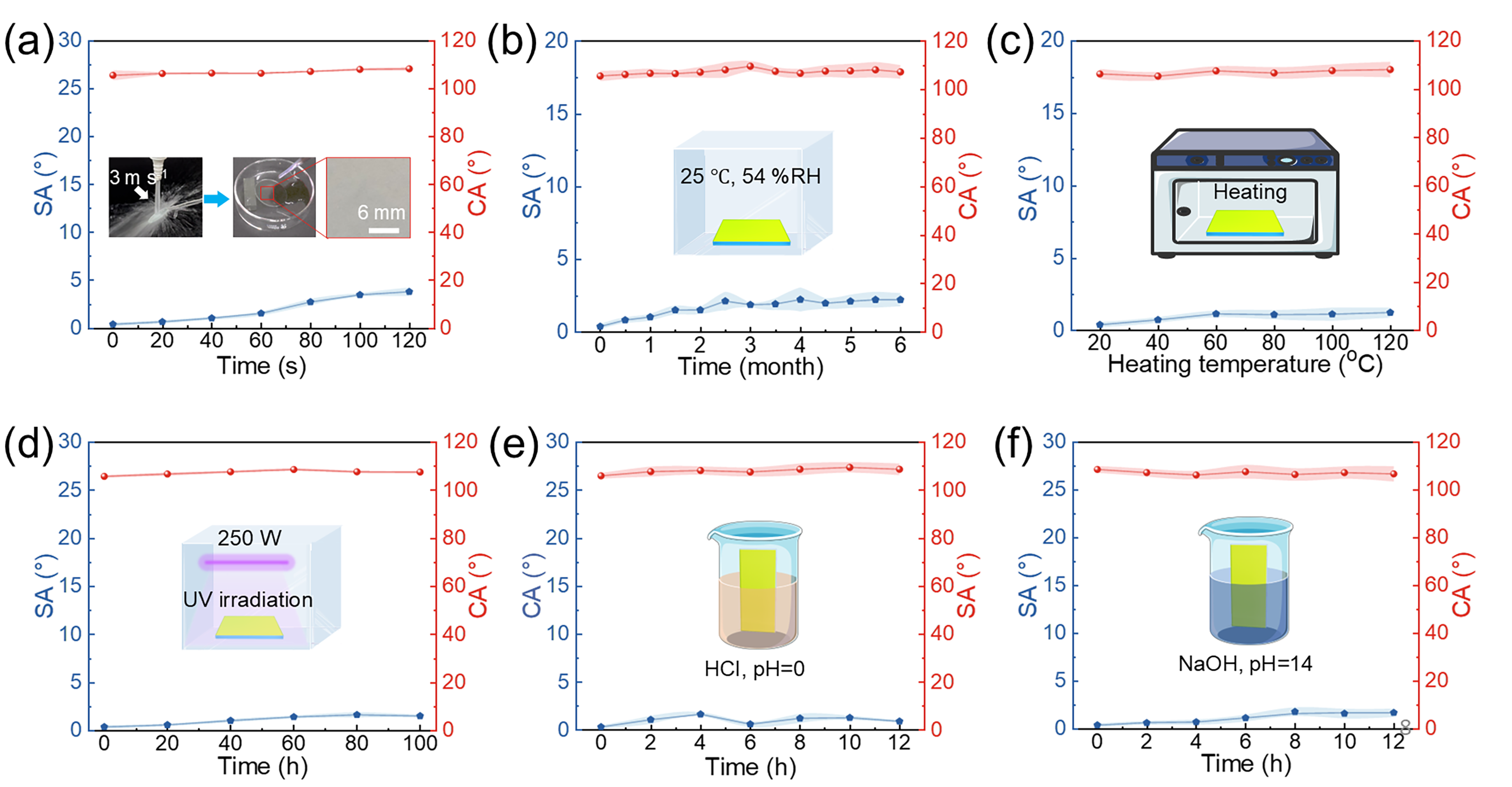


Figure S7. Effects of (a) rotation time in water, (b) storage time in air, (c) heating temperature, (d) UV irradiation time, (e,f) immersion time in strong acid and alkali solutions on the CA and SA.


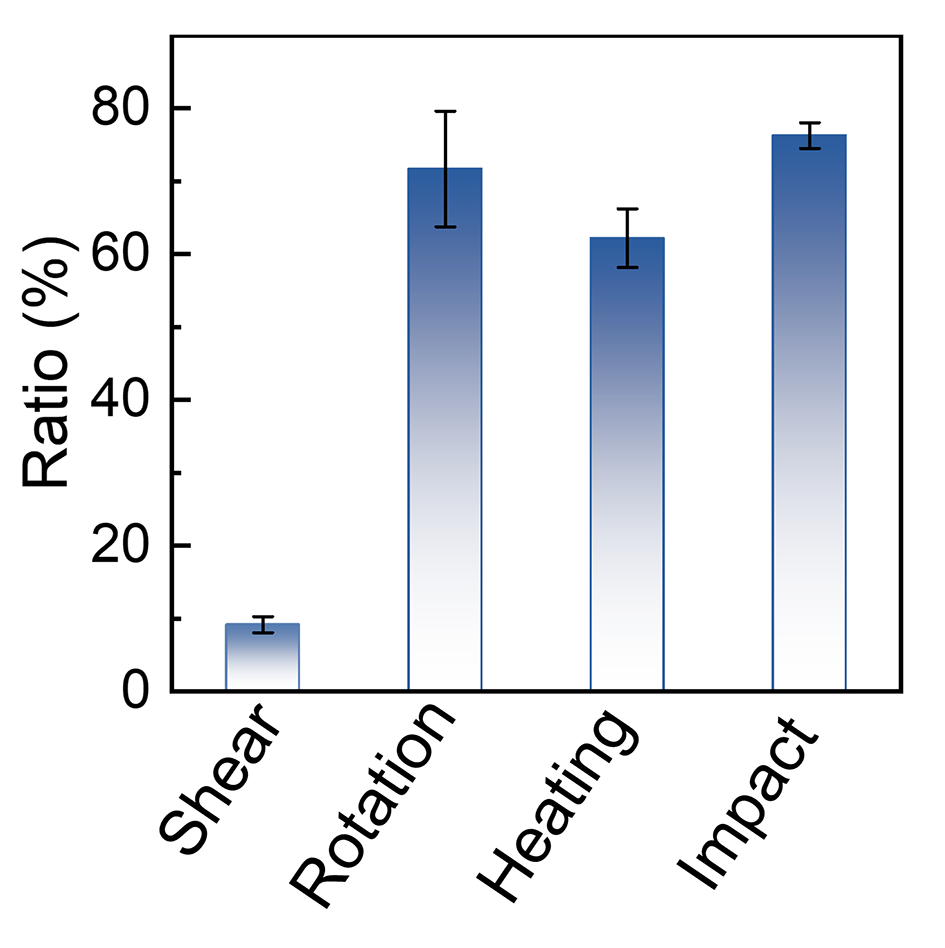


Figure S8. Loss rate of the SO after various tests.


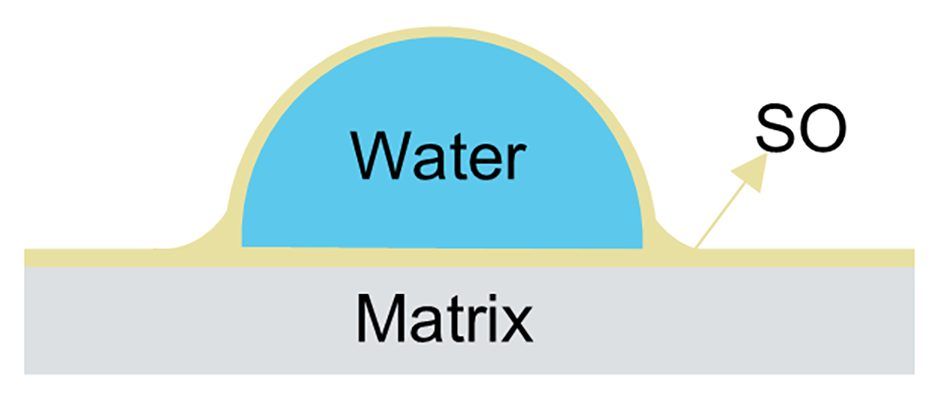


Figure S9. Schematic of “cloak” phenomenon.


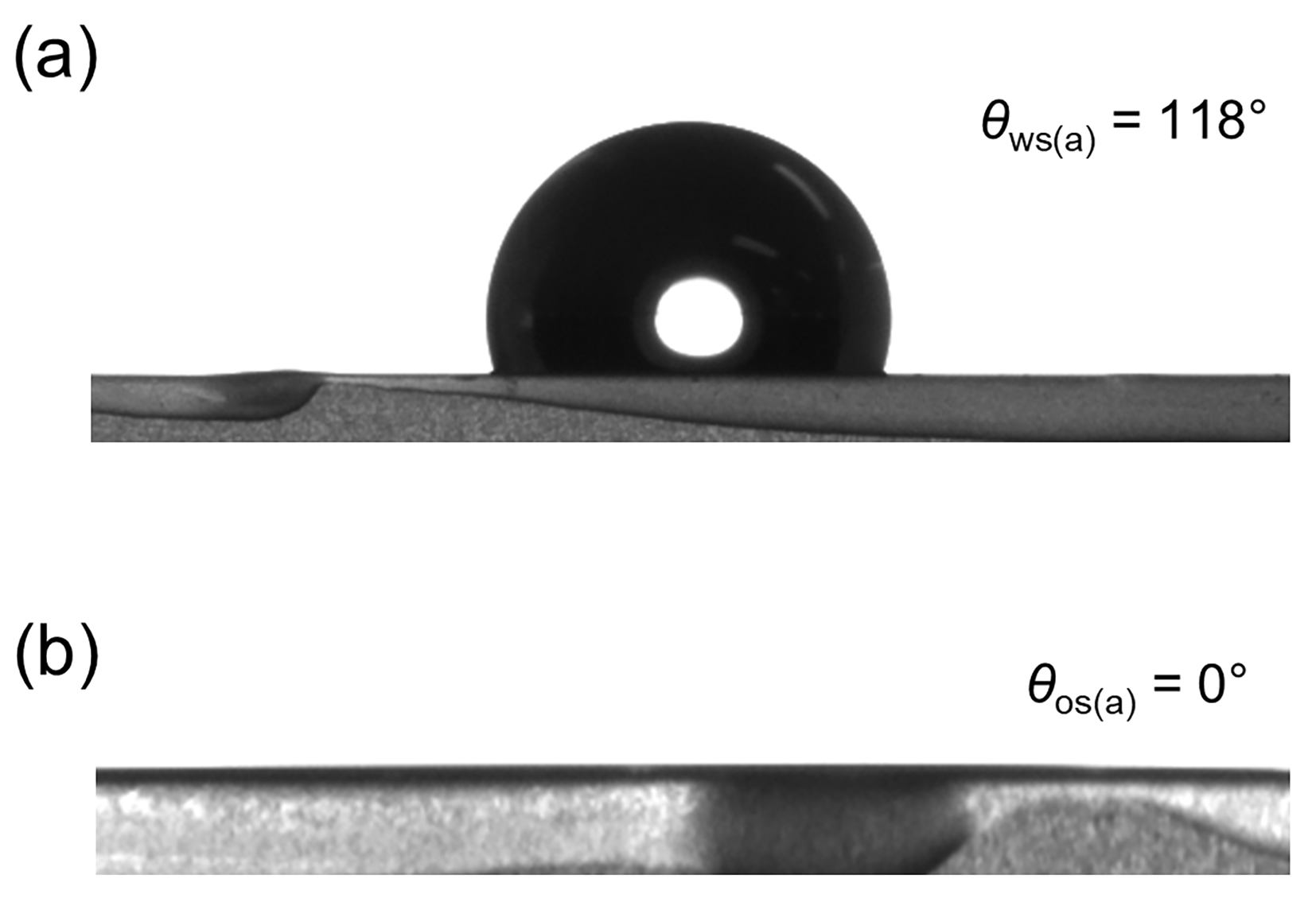


Figure S10. CA of different droplets on EINO. (a) water. (b) SO.


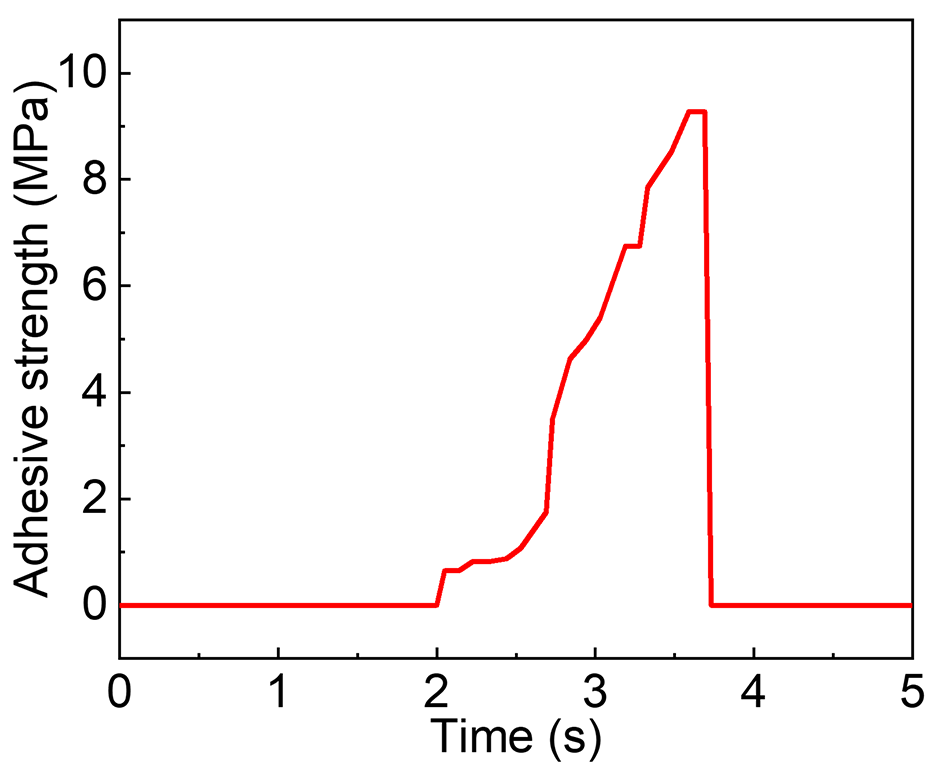


Figure S11. Variation in the adhesive strength between the EINO film and glass substrate with time after the high and low temperature cycling test.


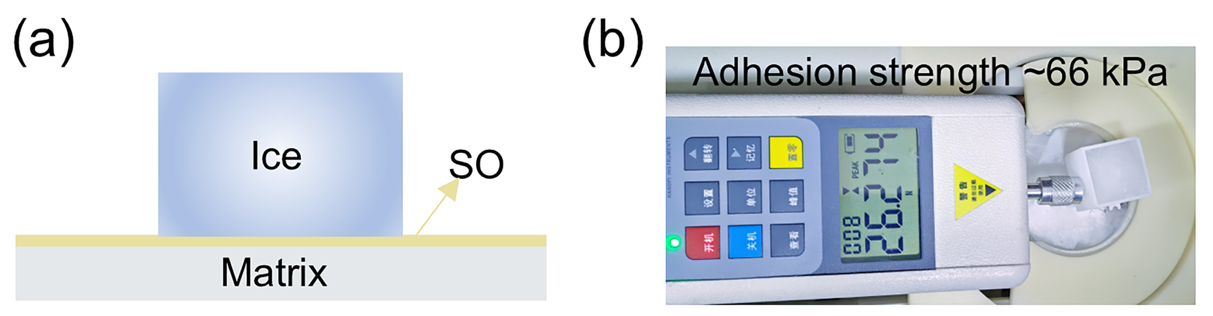


Figure S12. (a) Schematic showing the separation of ice from the film matrix through the unfrozen SO layer. (b) Adhesion strength of ice on EINO.


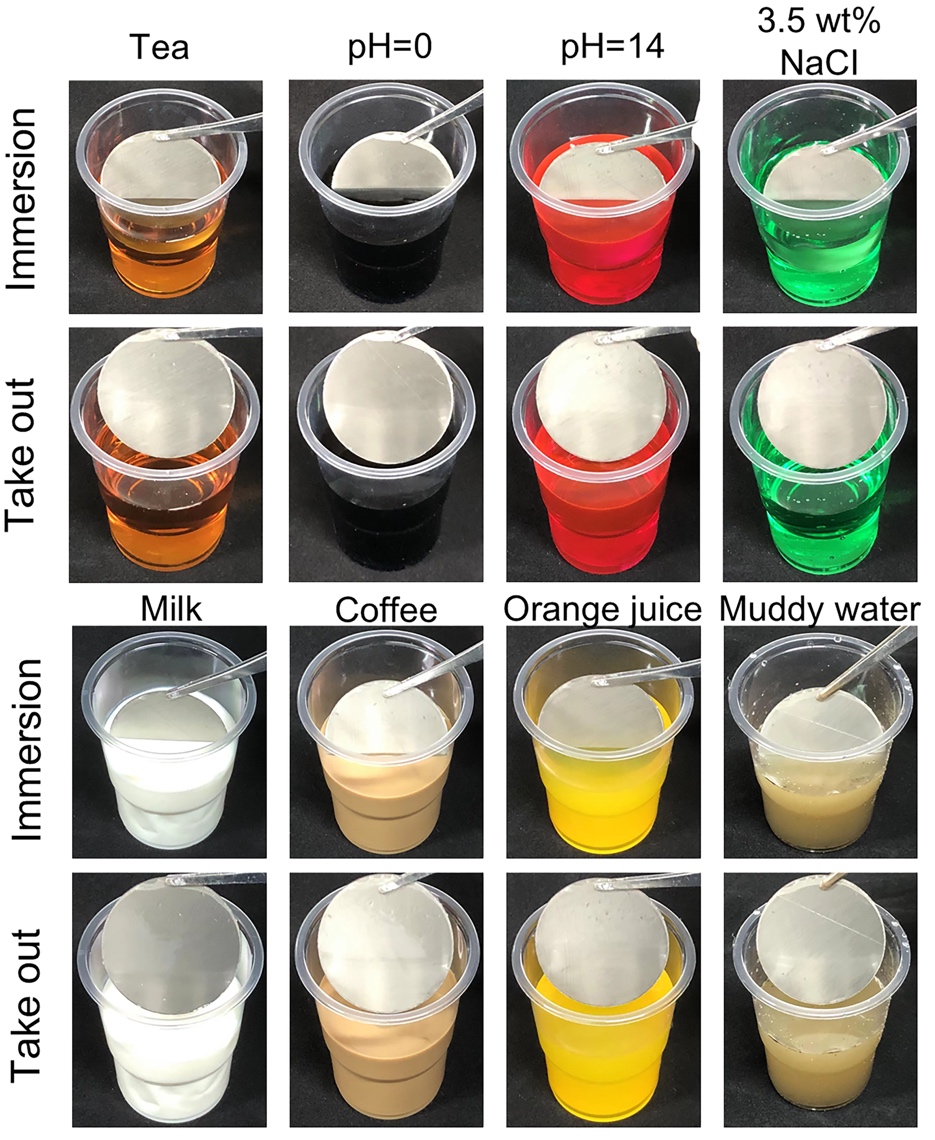


Figure S13. Images showing the self-cleaning property of the BCUSF immersed in various liquids, including acid, alkali, salt water, muddy water, coffee, and other drinks.


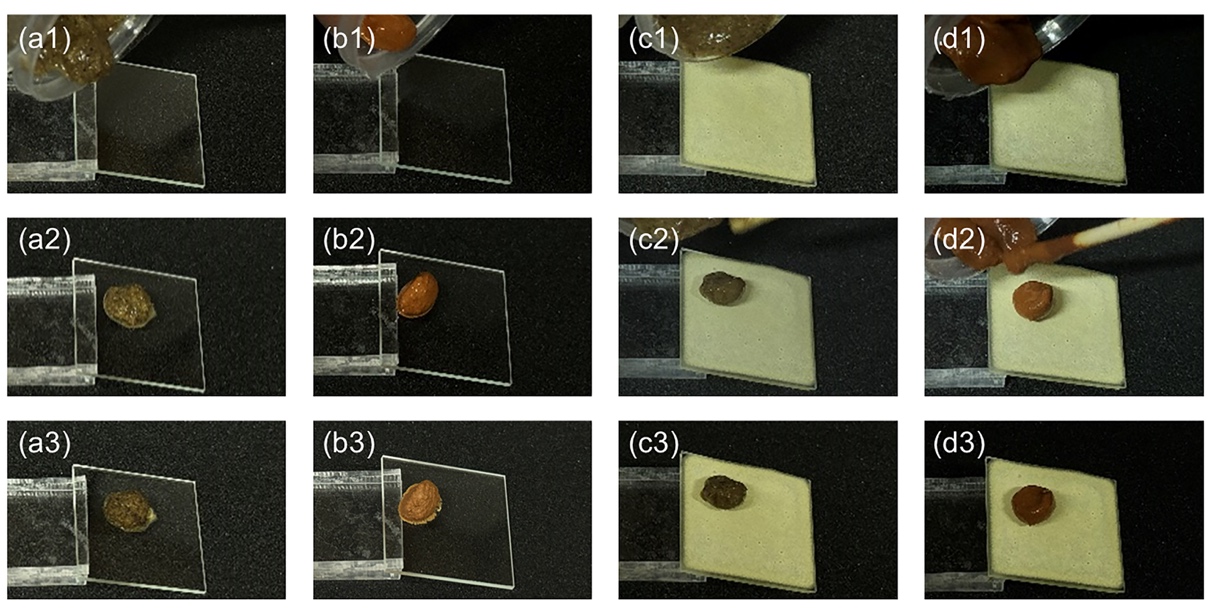


Figure S14. Self-cleaning tests of bare glass sheets and superhydrophobic surfaces. Sequential images of (a) bird droppings and (b) mud dropped on bare glass sheets. Sequential images of (c) bird droppings and (d) mud dropped on superhydrophobic surfaces.


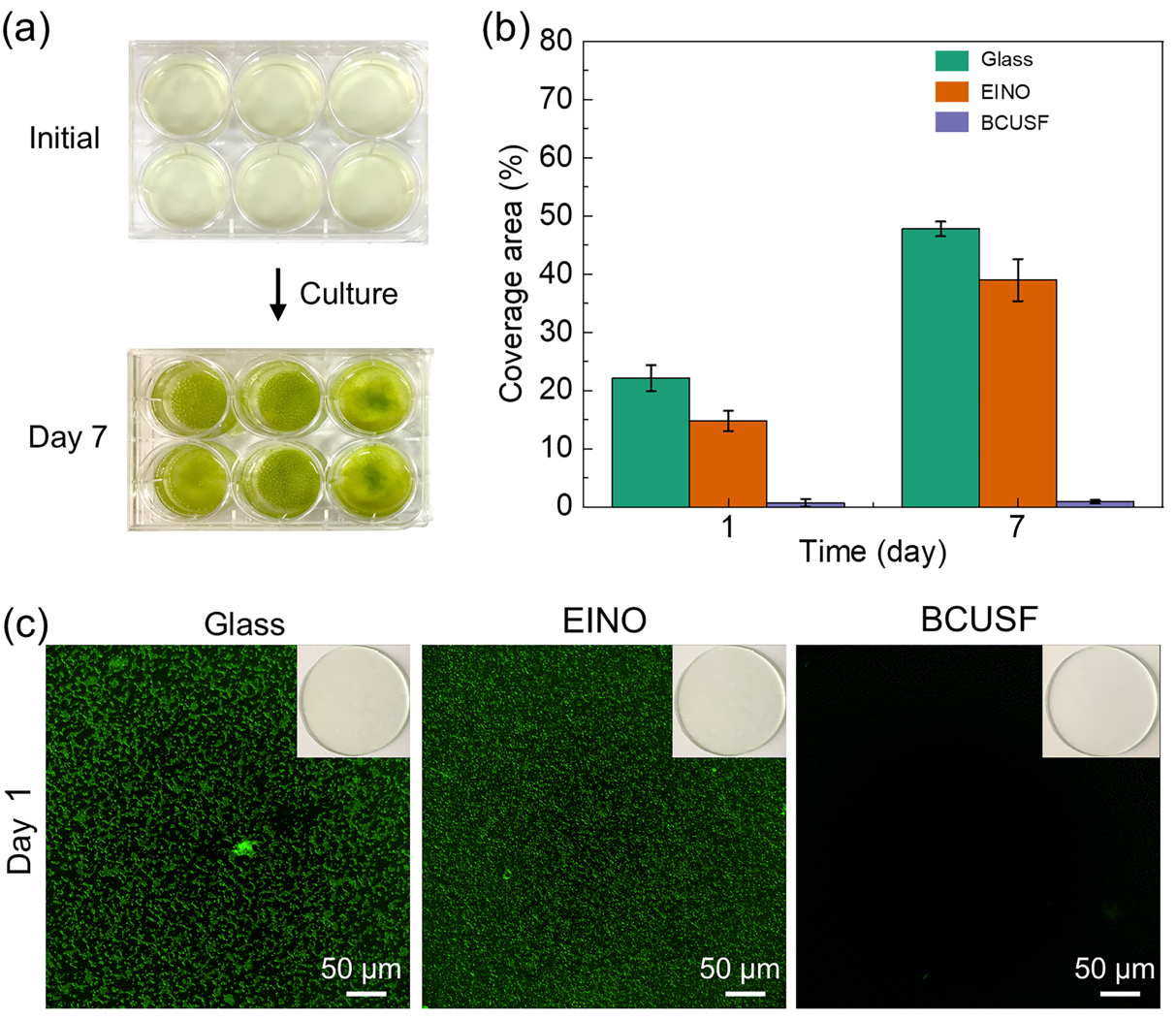


Figure S15. Anti-fouling tests of BCUSF. (a) Optical photographs of chlorella after 7 days of culture. (b) Effect of culture time on the coverage rate of chlorella on various samples. (c) Fluorescence images of various samples after being placed in chlorella solution for 1 day. The inset was the corresponding optical photographs.


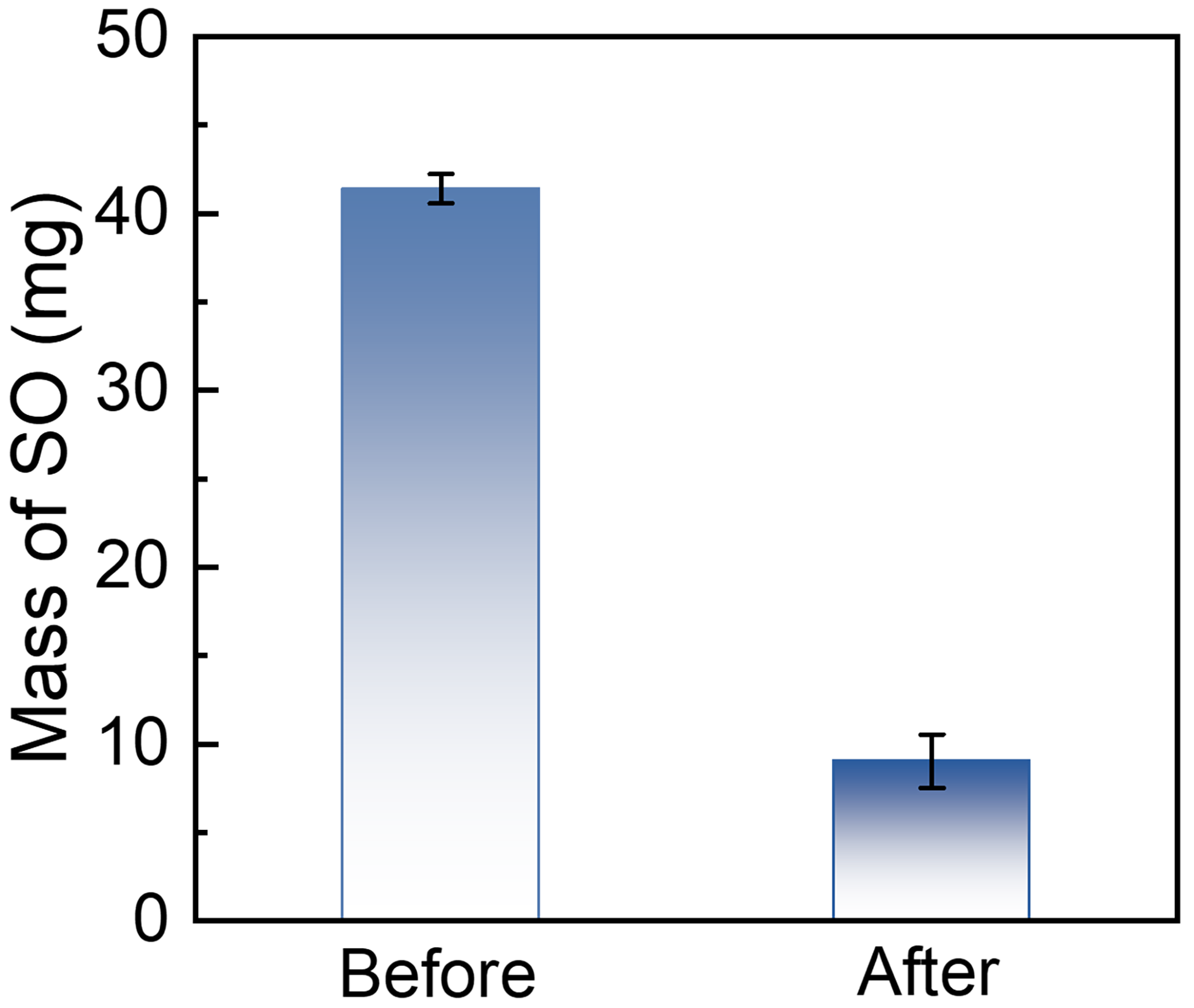


Figure S16. SO weight of the BCUSF before and after the 25 icing/de-icing cycles.


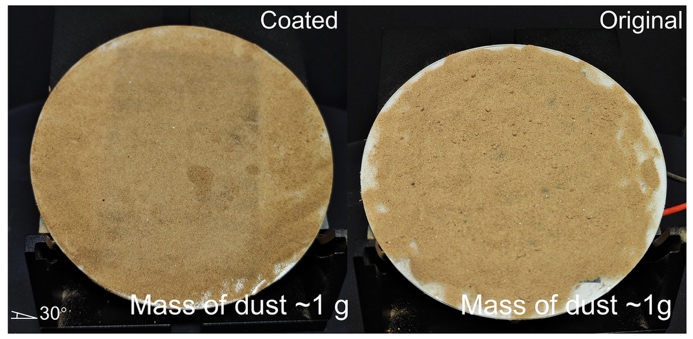


Figure S17. Optical photograph showing the adhesion of dust on the original and BCUSF-coated solar panels positioned at a tilt angle of 30°.


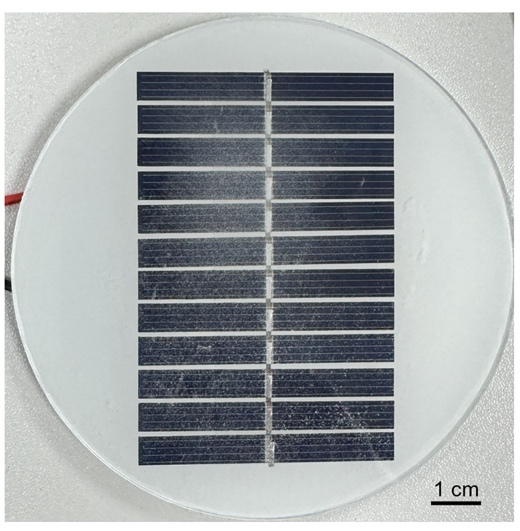


Figure S18. Optical photograph of an original solar panel after being impacted by sand for 600 s from a height of 35 cm at an angle of 30°.


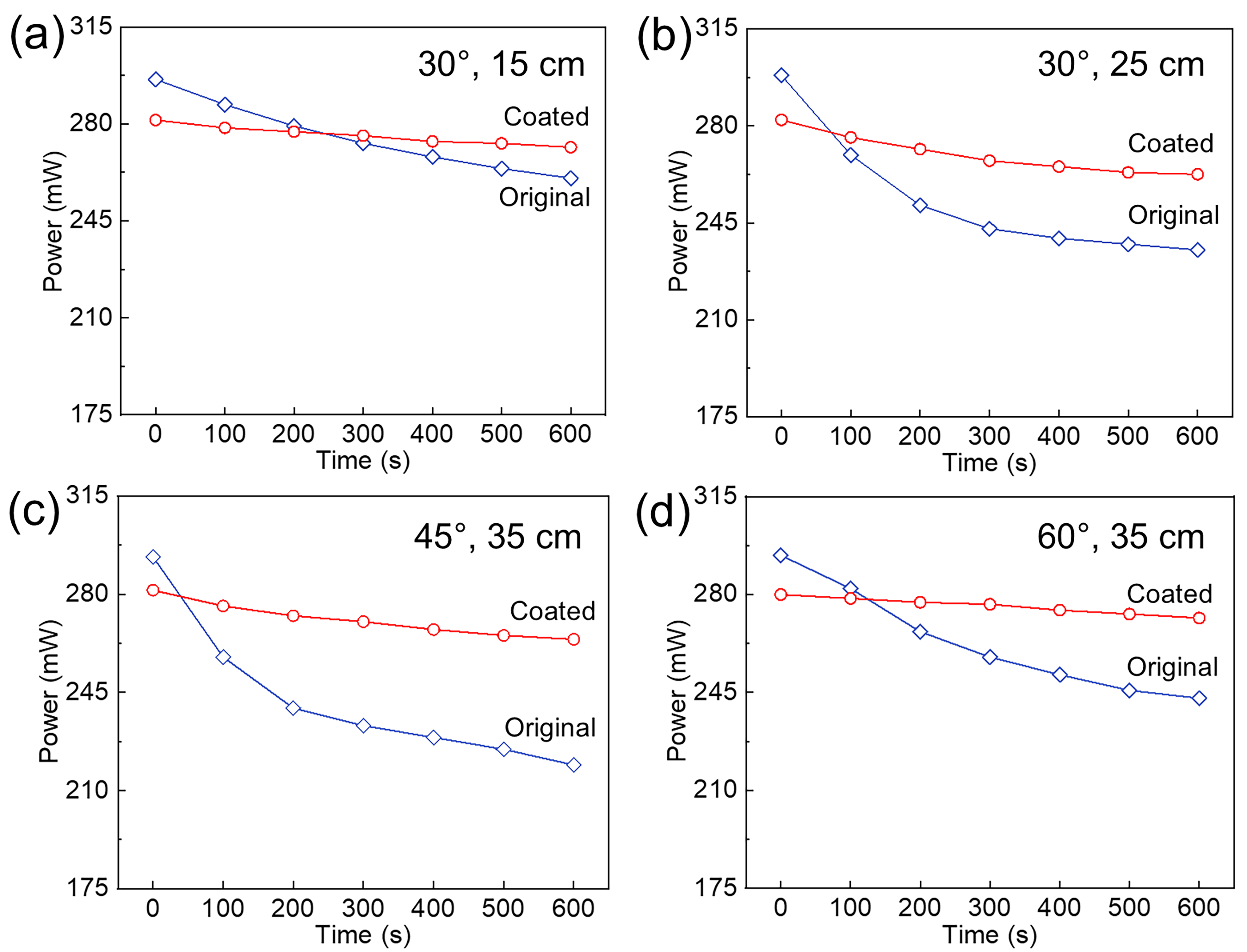


Figure S19. Effect of tilt angle and height on the output power of solar panels after falling sand impact. (a-d) Output power of original and BCUSF-coated solar panels as a function of impact time.


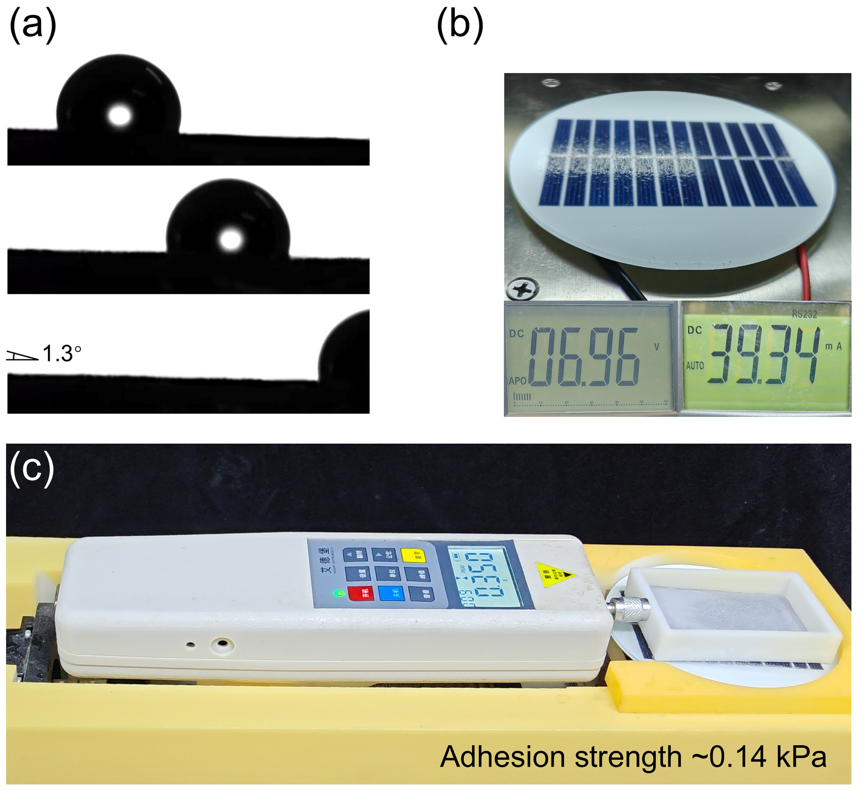


Figure S20. (a) SA, (b) output power, and (c) ice adhesion strength of the BCUSF-coated solar panel after aging for 168 h.


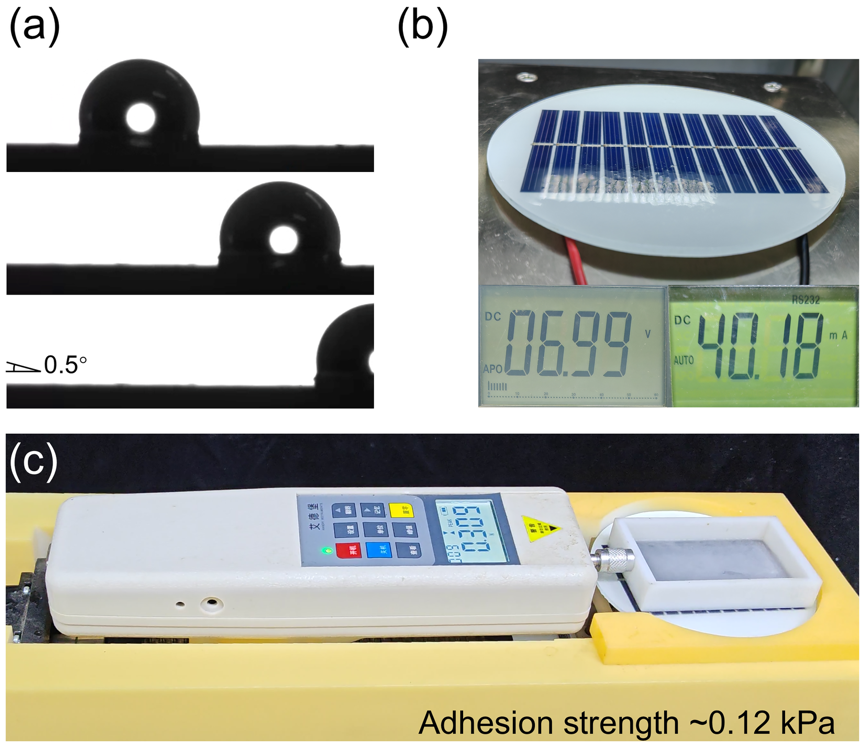


Figure S21. (a) SA, (b) output power, and (c) ice adhesion strength of the BCUSF-coated solar panel fabricated by the roller coating method.

**Supplementary Table**

Table S1. Raw material cost of the BCUSF.

| Product | Wholesale price | Recommended  painting area | Price per  square meter |
| --- | --- | --- | --- |
| EC | $67.12 per kg | 10 g per m^2^ | $0.67 |
| EA | $7.50 per kg | 60 g per m^2^ | $0.45 |
| IPES | $14.04 per kg | 12 g per m^2^ | $0.17 |
| OH-PDMS | $193.22 per kg | 1.5 g per m^2^ | $0.28 |
| NH_2_-PDMS | $193.22 per kg | 0.5 g per m^2^ | $0.09 |
| DBTDA | $57.57 per kg | 0.4 g per m^2^ | $0.02 |
| SO | $35.12 per kg | 0.4 g per m^2^ | $0.01 |
| Total | | | $1.69 |

**Supplementary Movies**

Movie S1. Sliding of a 5 μL water droplet on BCUSF.

Movie S2. Sliding of a 0.5 μL water droplet on BCUSF.

Movie S3. High-speed centrifugation test of BCUSF at 8000 r min^-1^.

Movie S4. Water-jet impacting test of BCUSF at 3 m s^-1^.

Movie S5. De-icing test on bare glass sheet.

Movie S6. De-icing test on BCUSF-coated glass sheet.

Movie S7. Self-cleaning test of BCUSF for resistance to dust contamination.

Movie S8. Self-cleaning test of BCUSF for resistance to mud contamination.

Movie S9. Self-cleaning test of BCUSF for resistance to bird droppings contamination.

Movie S10. De-icing test on original solar panel.

Movie S11. De-icing test on BCUSF-coated solar panel.
